# Dimensions of a Living Cochlear Hair Bundle

**DOI:** 10.3389/fcell.2021.742529

**Published:** 2021-11-25

**Authors:** Katharine K. Miller, Patrick Atkinson, Kyssia Ruth Mendoza, Dáibhid Ó Maoiléidigh, Nicolas Grillet

**Affiliations:** Department of Otolaryngology-Head and Neck Surgery, School of Medicine, Stanford University, Stanford, CA, United States

**Keywords:** stereocilia, hair cell, mechanotransduction, hair bundle, mouse, deafness, hearing loss, electron microscopy

## Abstract

The hair bundle is the mechanosensory organelle of hair cells that detects mechanical stimuli caused by sounds, head motions, and fluid flows. Each hair bundle is an assembly of cellular-protrusions called stereocilia, which differ in height to form a staircase. Stereocilia have different heights, widths, and separations in different species, sensory organs, positions within an organ, hair-cell types, and even within a single hair bundle. The dimensions of the stereociliary assembly dictate how the hair bundle responds to stimuli. These hair-bundle properties have been measured previously only to a limited degree. In particular, mammalian data are either incomplete, lack control for age or position within an organ, or have artifacts owing to fixation or dehydration. Here, we provide a complete set of measurements for postnatal day (P) 11 C57BL/6J mouse apical inner hair cells (IHCs) obtained from living tissue, tissue mildly-fixed for fluorescent imaging, or tissue strongly fixed and dehydrated for scanning electronic microscopy (SEM). We found that hair bundles mildly-fixed for fluorescence had the same dimensions as living hair bundles, whereas SEM-prepared hair bundles shrank uniformly in stereociliary heights, widths, and separations. By determining the shrinkage factors, we imputed live dimensions from SEM that were too small to observe optically. Accordingly, we created the first complete blueprint of a living IHC hair bundle. We show that SEM-prepared measurements strongly affect calculations of a bundle’s mechanical properties – overestimating stereociliary deflection stiffness and underestimating the fluid coupling between stereocilia. The methods of measurement, the data, and the consequences we describe illustrate the high levels of accuracy and precision required to understand hair-bundle mechanotransduction.

## Introduction

Hair bundles are the mechanosensory organelles of hair cells that detect forces induced by sound in auditory systems, head motion in vestibular systems, or fluid flow in lateral lines. The hair bundle consists of an assembly of stereocilia – cylindrical cellular protrusions with a beveled top and a tapered bottom filled with F-actin (Duvall et al., 1966; Mulroy, 1974; Flock and Cheung, 1977; DeRosier et al., 1980; Tilney et al., 1980; Tilney and Saunders, 1983; Kaltenbach et al., 1994). Stereocilia are arranged in rows of increasing height, forming a staircase (Tilney and Saunders, 1983; Tilney et al., 1988). A single microtubule-based cilium, called the kinocilium, is connected to the stereocilia at a central location behind the tallest row during development, and is maintained or eliminated in mature hair cells depending on the species and sensory system (Wersall, 1956; Roth and Bruns, 1992). External nanofilaments connect the stereocilia, including tip links that connect the tip of a stereocilium to its taller neighbor (Osborne et al., 1984; Pickles et al., 1984). Mechanical deflection of the hair bundle toward its tallest row extends gating springs, composed of tip links and other elements, which convey forces that modulate mechanotransducer-channel open probability, which in turn drives the hair-cell receptor potential via the influx of potassium ions into the cell (Davis, 1965; Howard and Hudspeth, 1988; Bartsch et al., 2019; Ó Maoiléidigh and Ricci, 2019).

In the rodent auditory organ, the cochlea, hair-bundle development begins embryonically. Hair bundles form a staircase by birth, acquire mechanosensitivity at P0-P1, and reach maturity after P21 (Lim and Anniko, 1985; Roth and Bruns, 1992; Kaltenbach et al., 1994; Zine and Romand, 1996; Waguespack et al., 2007; Lelli et al., 2009; Kim and Fettiplace, 2013; Pan et al., 2013; Beurg et al., 2018; Krey et al., 2020; Trouillet et al., 2021). From P0-P21, hair-bundle morphology changes drastically, with stereocilia increasing or decreasing in height and width depending on their row (Tilney et al., 1980; Tilney and DeRosier, 1986; Roth and Bruns, 1992; Kaltenbach et al., 1994; Krey et al., 2020). Stereociliary height and hair-bundle morphology stabilize in adulthood.

Each mature hair bundle has a distinct number of stereocilia with defined dimensions depending on the species, sensory organ, position within their organ, hair-cell type (e.g., inner hair cells, outer hair cells (OHCs), or vestibular hair cells), and row within a bundle (Wright, 1984; Kaltenbach et al., 1994; Zine and Romand, 1996; Ricci et al., 1997; Xue and Peterson, 2006; Xiong et al., 2012; Yarin et al., 2014). For example, mature apical rodent cochlear hair bundles are taller than basal hair bundles and have fewer stereocilia per row (Garfinkle and Saunders, 1983; Lim, 1986; Roth and Bruns, 1992; Kaltenbach et al., 1994). Although genetic mutations that cause hearing loss often affect the number of stereocilia and their dimensions, their effects on hair-bundle mechanics are not well-understood (Petit and Richardson, 2009; Richardson and Petit, 2019).

Stereociliary dimensions determine the mechanical response of a hair bundle to a stimulus: for example, a stereocilium’s height determines its stiffness, and the geometrical relationship between neighboring stereocilia determines their coupling by fluid and the gating-spring extension in response to stereociliary deflection (Howard and Ashmore, 1986; Howard and Hudspeth, 1988; Crawford et al., 1989; Jacobs and Hudspeth, 1990; Geisler, 1993; Pickles, 1993; Zetes, 1995; Furness et al., 1997; Zetes and Steele, 1997; Karavitaki and Corey, 2010; Baumgart, 2011; Kozlov et al., 2011; Hadi et al., 2020). Determining a hair bundle’s mechanical properties is challenging experimentally and often relies on mathematical models of the hair bundle (Crawford and Fettiplace, 1985; Howard and Hudspeth, 1987; Kozlov et al., 2011; Powers et al., 2012, 2014; Ó Maoiléidigh and Hudspeth, 2013; Nam et al., 2015; Gianoli et al., 2017; Milewski et al., 2017; Cartagena-Rivera et al., 2019; Tobin et al., 2019). However, models require prior knowledge of stereociliary dimensions. If these measurements have not been determined for living hair bundles, models either make assumptions or use values obtained from electron microscopy (EM) (Furness et al., 1997; Smith and Chadwick, 2011; Zetes et al., 2012; Ó Maoiléidigh and Hudspeth, 2013; Nam et al., 2015).

While EM can achieve sub-nanometer resolution, the preparation method is deleterious to the tissue: the tissue is strongly fixed with glutaraldehyde (and in some cases further post-fixed with osmium tetroxide) and dehydrated in successive ethanol baths. Samples are then either embedded in a resin to generate thin sections imaged by Transmission EM (TEM), or dried in a critical-point drying chamber, after replacement of ethanol by liquid-CO2, coated with a thin metal layer, and observed by SEM (Bozzola and Russell, 1992). These EM sample preparation steps induce dimensional distortions. TEM samples are less subject to these distortions due to the presence of a supporting liquid surrounding the sample until the resin hardens, whereas all fluids are removed during SEM sample drying (Nordestgaard and Rostgaard, 1985). A major limitation of TEM, however, is that it produces clear results only for small numbers of cells, because capturing structures of interest within a TEM section is difficult. In comparison, conventional SEM allows direct imaging of the entire ultrastructure of a large number of cells, but the sample preparation induces substantial tissue shrinkage. Therefore, stereociliary dimensions obtained by SEM are underestimated to a large extent, but the magnitude of this shrinkage has not been well-quantified (Jensen et al., 1981). Still, SEM has been valuable for performing relative comparisons, such as between samples of different genotypes or between groups undergoing different treatments (Hunter-Duvar, 1978; Holme and Steel, 2002; Gagnon et al., 2006; Webb et al., 2011; Xiong et al., 2012; Lelli et al., 2016; Vélez-Ortega et al., 2017; Trouillet et al., 2021).

In addition to SEM, stereociliary dimensions have been measured in mildly-fixed samples – incubating in paraformaldehyde for 30 min, permeabilizing the tissue, labeling the actin-core of stereocilia using fluorescently labeled phalloidin, and finally imaging with fluorescence microscopy. With conventional light microscopy, stereociliary dimensions can be determined with a lateral resolution of about 200 nm and with recent technological improvements in super-resolution fluorescence microscopy, the resolution can be further improved (Schermelleh et al., 2019; Krey et al., 2020). However, it remains unclear whether mild paraformaldehyde fixation affects these measurements.

Live stereociliary dimensions have rarely been determined, resulting in limited information about the live morphology of different types of hair bundles. Because the available live-cell studies used different techniques and the imaged hair bundles differ greatly in their morphology, this data cannot be combined to create a complete description of a given hair bundle. These studies include: fluorescent imaging of overexpressed actin-EGFP in P2-P5 mouse utricular stereocilia (Drummond et al., 2015), fluorescent labeling of the stereociliary membrane with a lipophilic dye in P8-P9 rat IHCs (George et al., 2020), light-microscopy of isolated vestibular bullfrog hair cells (Jacobs and Hudspeth, 1990) or P7-P10 rat IHC hair bundles lying flat on the apical hair-cell surface (Tobin et al., 2019), and scanning ion conductance microscopy of the surface of P4 rat IHC hair bundles (Vélez-Ortega and Frolenkov, 2016; Galeano-Naranjo et al., 2021). In summary, a complete set of stereociliary heights, widths, and separations has still not been determined for living hair bundles. Moreover, the size differences between live, mildly-fixed, and SEM-processed preparations remain unknown. Determining scaling factors between preparations will allow us to impute live stereociliary dimensions from fixed preparations, which will be especially useful for rare samples such as human hair cells (Wright, 1981, 1984; Jeffries et al., 1986; Lavigne-Rebillard and Pujol, 1986, 1987, 1990).

To address the question of living hair-bundle dimensions, we focused on the mouse – the mammalian genetic animal model for inherited hearing loss. We accurately measured the stereociliary height, width, and separation of apical IHCs from P11 littermate C57BL/6J mice imaged under live, mildly-fixed, or SEM-prepared conditions. We found that live and mildly-fixed bundles have similar stereociliary dimensions, while SEM preparation reduced all stereociliary dimensions by similar amounts. Using the shrinkage factors for SEM, we were able to impute live dimensions that were too small to be measured optically. We also show how calculations of stereociliary stiffness, fluid coupling between stereocilia, and the geometric relationship between gating-spring extension and stereociliary deflection are affected when SEM-determined measurements are used instead of live dimensions.

## Results

### Live and Mildly-Fixed Apical Inner-Hair-Cell Stereocilia Have Similar Heights

**FIGURE 1:**
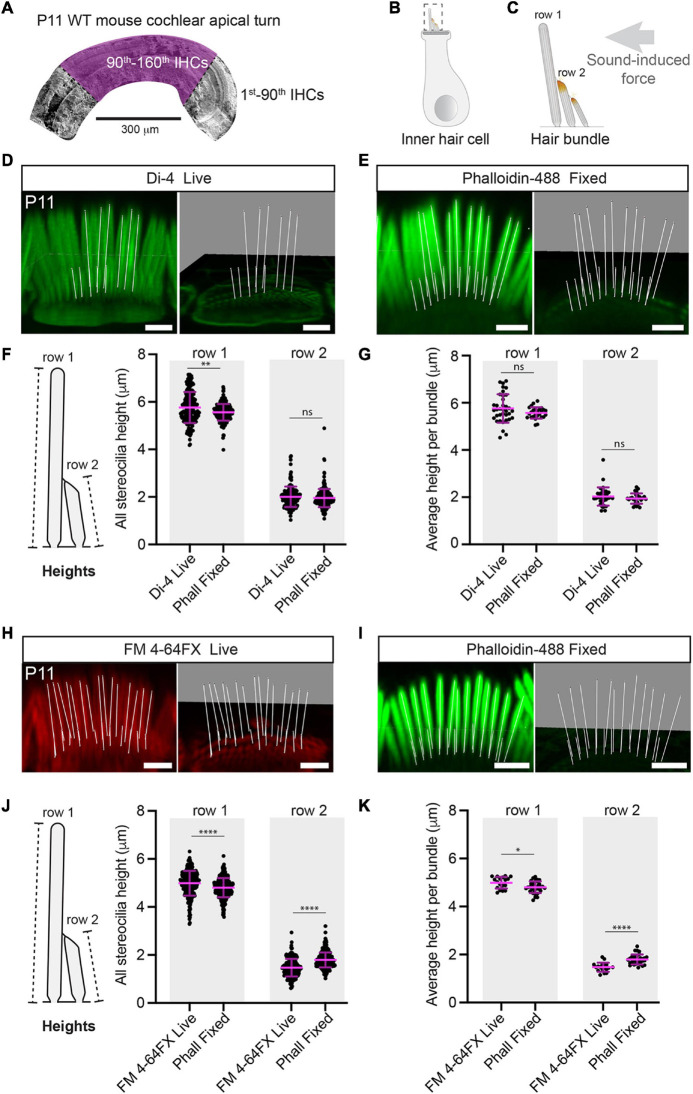
Stereociliary heights are the same in live-stained and mildly-fixed conditions. **(A)** Hair-bundle dimensions were measured in a spatially well-defined segment of the P11 WT apical cochlea, highlighted here in purple on an SEM micrograph. The number of inner hair cells (IHCs) are indicated before and within the segment of interest. **(B)** A hair bundle is shown protruding from the apical surface of an inner hair cell. **(C)** A cochlear hair bundle consists of stereocilia arranged in rows of graded height, which are deflected by sound-induced forces, leading to the opening of mechanosensitive ion channels at the tips of rows 2 and 3. Scale bars: 2 μm. Representative 3-D reconstructed images are shown for Di-4 live **(D)** and phalloidin-488 mildly-fixed **(E)** bundles. Lines (white) represent the height measurements for row 1 (tallest) and row 2 stereocilia and are shown with and without the stereociliary volumes. Scale bars: 2 μm. **(F)** Stereociliary heights are shown from P11 WT IHCs live-stained with Di-4 (row 1: 195 stereocilia, row 2: 165 stereocilia, 3 cochleae, 3 animals) or with phalloidin after mild fixation (row 1: 219 stereocilia, row 2: 240 stereocilia, 3 cochleae, 3 animals). Error bars represent the mean ± SD. Di-4 and phalloidin heights differ statistically for row 1 (Mann-Whitney U tests were used here and in figures hereafter, *P* = 0.0027, percentage difference = –4 ± 13% relative to Di-4) and but not for row 2 (*P* = 0.22). These small (magnitude ≤ 10%) and highly uncertain (magnitudes similar to or smaller than their SDs) percentage differences are unlikely to be biologically important, biological importance cannot be ascribed to highly uncertain percentage differences. **(G)** Di-4 and phalloidin average stereociliary heights per bundle do not differ statistically for row 1 and row 2 (36 hair bundles, 3 cochleae, 3 animals for Di-4; 26 hair bundles, 3 cochleae, 3 animals for phalloidin; row 1: *P* = 0.21, row 2: *P* = 0.32). Representative 3-D reconstructed images are shown for FM 4-64FX live **(H)** and phalloidin-488 mildly-fixed **(I)** IHC hair bundles from a slightly less mature P11 WT litter. Lines (white) represent the height measurements and are shown with and without stereociliary volumes. Scale bars: 2 μm. **(J)** Stereociliary heights are shown from slightly less mature P11 WT IHCs live-stained with FM 4-64FX (row 1: 419 stereocilia, row 2: 408 stereocilia, 2 cochleae, 2 animals) or phalloidin after mild fixation (row 1: 233 stereocilia, row 2: 224 stereocilia, 2 cochleae, 2 animals). FM 4-64FX and phalloidin heights differ statistically for row 1 and for row 2 (*P* < 0.0001 for both, row 1 percentage difference = –4 ± 13% relative to FM 4-64FX, row 2 percentage difference = 22 ± 33% relative to FM 4-64FX), but the differences are small and/or highly uncertain, implying no biological importance. **(K)** Average stereociliary heights per bundle are shown for row 1 and row 2 using FM 4-64FX (18 hair bundles, 2 cochleae, 2 animals) and phalloidin (33 hair bundles, 2 cochleae, 2 animals). Although row 1 and 2 heights are statistically different between conditions, their percentage differences are small and/or highly uncertain, implying no biological importance (row 1: *P* = 0.017, percentage difference = –4 ± 7% relative to FM 4-64FX; row 2: *P* < 0.0001, percentage difference = 22 ± 19% relative to FM 4-64FX. Horizontal lines indicate comparisons using the Mann-Whitney U test: ns *P* > 0.05, ^∗^*P* < 0.05, ^∗∗^*P* < 0.01, ^∗∗∗^*P* < 0.001, ^****^P < 0.0001.

Hair-bundle investigations are typically performed in the mouse from P0 to P11, when the cochlear bone can be removed with less damage to the hair cells than at older ages (Kim and Fettiplace, 2013; Asai et al., 2018; Corns et al., 2018; Trouillet et al., 2021). For comparison with previous studies, we imaged and measured stereociliary dimensions in C57BL/6J wild-type (WT) mice at postnatal day (P) P11. We focused on IHCs in the apical cochlear turn, because their stereocilia are tall and wide (Garfinkle and Saunders, 1983). To avoid the heterogeneity found within the most apical IHCs, we focused on hair bundles from the 90th to 160th IHCs from the apex (Figure 1A). This is 7–20% of the cochlear length, measured from the apex, corresponding to the 5–8.5 kHz characteristic-frequency range in adults (Müller et al., 2005). At P11, the tallest IHC stereociliary row (row 1) is 2–3 times taller than the second row (row 2), which facilitated measurements (Figures 1B,C). To further reduce measurement variability due to maturation differences, we used animals from a single litter in each experiment. To preserve hair-cell viability, apical cochlear turns were dissected in the extracellular solution used for mechanotransduction electrophysiological recordings (George et al., 2020). We stained individual cochleae from a single animal in one of two ways. One cochlea was live-stained for 5 min with a lipophilic dye that becomes strongly fluorescent upon binding to cell membranes. Imaging was performed immediately and for a maximum duration of 37 min using a confocal microscope in Airyscan mode equipped with an immersion lens (Figure 1D). The other cochlea was mildly fixed (30 min in 4% paraformaldehyde at room temperature), permeabilized, stained with phalloidin-Alexa488, which labels the stereociliary actin-core, and imaged immediately after the corresponding live sample using the same optical settings and lens (Figure 1E). Hair-bundle image stacks were reconstructed and analyzed as 3-D objects with Imaris (Oxford Instruments) software. Stereocilium heights from row 1 and row 2 were measured by manually placing measurements points at the stereociliary bases and tops in 3-D space. We defined the top as the location where the fluorescence signal suddenly decayed, and the base as the narrow end of stereociliary taper (Supplementary Movies 1–3). For comparison with SEM (see below), we focused on fully visible stereocilia, or row 1 stereocilia abutting a fully visible row 2 stereocilium, excluding the stereocilia at row edges. All measurements are presented to the nearest 0.01 μm ± standard deviation (SD).

In our first set of experiments, we stained the IHC stereociliary membrane with Di-4-ANEPPDHQ (Di-4) lipophilic vital dye and compared measurements with mildly-fixed bundles stained with phalloidin-Alexa488. The height of row 1 stereocilia was 5.76 ± 0.65 μm in the Di-4 live condition and 5.56 ± 0.35 μm in the mildly-fixed condition (Figure 1F and Supplementary Figure 1A). The live and mildly-fixed row 1 heights were statistically different due to the large number of samples (because the data was rarely normal, the Mann-Whitney U test was used here and hereafter for comparisons unless stated otherwise; *P* = 0.0027), but the percentage difference was small (magnitude ≤ 10%) and highly uncertain (magnitude similar to or smaller than its SD) (percentage difference = −4 ± 13% relative to Di-4) (Figure 1F). Small percentage differences are unlikely to be biologically important and we cannot ascribe biological importance to highly uncertain percentage differences (Hughes and Hase, 2010). Row 2 stereociliary heights from the Di-4 live condition were also similar to the mildly-fixed condition (Di-4: 2.00 ± 0.43 μm, mildly-fixed 1.95 ± 0.38 μm, *P* = 0.22) (Figure 1F). Average stereociliary heights per hair bundle were not statistically different between conditions (row 1: 5.76 ± 0.60 μm for Di-4 vs. 5.57 ± 0.24 μm for mildly-fixed, *P* = 0.21; row 2: 2.03 ± 0.39 μm for Di-4 vs. 1.95 ± 0.22 μm for mildly-fixed, *P* = 0.32) (Figure 1G and Supplementary Figure 1B).

To confirm the results obtained with Di-4, we performed a second set of experiments using a chemically unrelated vital fluorescent lipophilic dye, FM 4-64FX (Thermo Fisher Scientific, F34653) and phalloidin-Alexa488 (Figures 1H,I and Supplementary Movie 3). As with Di-4, FM 4-64FX emits fluorescence when integrated into the membrane, but not when in solution. With another WT C57BL/6J mouse litter (P11 but earlier in development than the first), row 1 stereociliary height was 4.99 ± 0.52 μm for FM 4-64FX and 4.80 ± 0.40 μm for the mildly-fixed condition (Figure 1J and Supplementary Figure 1C). As with Di-4, while the heights between conditions were statistically different (*P* < 0.0001, percentage difference = 4 ± 13% relative to FM 4-64FX), the percentage difference was too small and uncertain to be biologically important (Figure 1J). Row 2 stereociliary heights in the FM 4-64FX live condition were statistically different to the mildly-fixed condition, but the percentage difference was too uncertain to be ascribed biological importance (FM 4-64FX live: 1.47 ± 0.37 μm, mildly-fixed 1.79 ± 0.31 μm, *P* < 0.0001, percentage difference = 22 ± 33% relative to FM 4-64FX) (Figure 1J). Similar results were found when comparing the average stereociliary heights per hair bundle: both row 1 and row 2 stereociliary heights were statistically different between conditions, but the row 1 percentage difference was small, and the row 2 percentage difference was highly uncertain (row 1: 4.99 ± 0.24 μm, row 2: 1.48 ± 0.19 μm for FM 4-64FX live vs. row 1: 4.81 ± 0.24 μm; row 2: 1.80 ± 0.21 μm for mildly-fixed; row 1, percentage difference = −4 ± 7% relative to FM 4-64FX, *P* = 0.017; row 2, percentage difference = 22 ± 19% relative to FM 4-64FX, *P* < 0.0001) (Figure 1K and Supplementary Figure 1D). In summary, the stereociliary heights measured in live conditions with vital lipophilic dyes were comparable to the heights measured after mild fixation and phalloidin staining, implying that either technique can be used to determine the heights of living stereocilia.

To establish how stereociliary height varies within a given hair bundle, we determined the coefficient of variation for rows 1 and 2 of each hair bundle (Supplementary Figures 2A,B). These data indicate low variability within each bundle. However, we noticed outlier heights within each bundle (Supplementary Figure 1), which we investigated by determining row 1 stereociliary heights relative to their position within the row using our mildly-fixed phalloidin samples (FM 4-64X litter). Each stereocilium was numbered relative to the central stereocilium (see below for the definition) (Supplementary Figure 2C). Stereocilia at position 1 were statistically taller than the central and the last stereocilia, but percentage differences were highly uncertain (position 0–1 percentage difference = −11 ± 14% relative to position 1, position 1-last percentage difference = −19 ± 17% relative to position 1; *P* < 0.0001 for both) (Supplementary Figures 2D,E). Although there were outliers within each bundle, these outliers did not occur at systematic positions within a row.

### Live-Stained Apical Inner-Hair-Cell Stereocilia of Slightly Different Ages Have Different Heights but Similar Widths

Less than a 1-day difference in age can cause large differences in stereociliary height, as can be seen in our data above. Although both litters were P11, the litter used for the Di-4 comparison (Litter 1) appeared to be slightly more mature than that the one used for the FM 4-64FX comparison (Litter 2). In the mildly-fixed samples from the two litters, there is a large difference in the height of row 1 stereocilia (Litter 1 (Di4): 5.56 ± 0.35 μm, Litter 2 (FM 4-64FX): 4.80 ± 0.40 μm, *P* < 0.0001, percentage difference = −14 ± 10% relative to Litter 1). A similar large difference in height is seen when comparing the two litters using the live-stained conditions (Litter 1 (Di4): 5.77 ± 0.65 μm, Litter 2 (FM 4-64FX): 4.99 ± 0.52 μm, *P* < 0.0001, percentage difference = −13 ± 15% relative to Litter 1) (Supplementary Figure 3). Although the heights differ greatly between litters, the percentage differences are highly uncertain. For the mildly-fixed averages per bundle, the height difference between litters is large and the percentage difference has low uncertainty (Litter 1 (Di4): 4.81 ± 0.24 μm, Litter 2 (FM 4-64FX): 5.56 ± 0.24 μm, *P* < 0.0001, percentage difference = −14 ± 6% relative to Litter 1). However, for the live-stained averages per bundle, the heights are statistically different but the percentage difference is highly uncertain (Litter 1 (Di4): 4.99 ± 0.24 μm, Litter 2 (FM 4-64FX): 5.76 ± 0.60 μm, *P* < 0.0001, percentage difference = −13 ± 11% relative to Litter 1).

**FIGURE 2:**
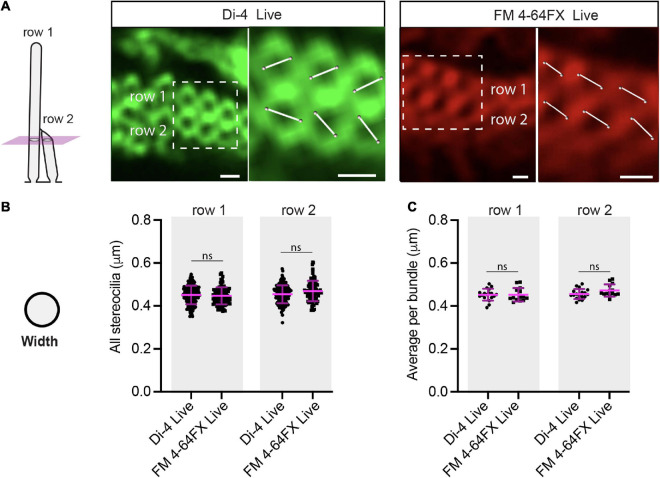
Stereociliary widths are the same in Di-4 and FM 4-64FX live-stained bundles of slightly different ages. **(A)** Virtual sections through 3-D reconstructed live-stained hair bundles below the row-2 tips show that stereociliary membranes form rings. Because rings look like distorted ovals, we determined a stereocilium’s width in 3-D by measuring the length of the shortest line passing through the ring’s center, with endpoints placed on the perimeter’s midsection. Scale bars: 0.5 μm. **(B)** No statistical difference was found for row 1 or row 2 stereociliary widths between Di-4 (row 1: 172 stereocilia, row 2: 149 stereocilia, 3 cochleae, 3 animals) and FM 4-64FX conditions (row 1: 101 stereocilia, row 2: 90 stereocilia, 2 cochleae, 2 animals) (row 1: *P* = 0.23, row 2: *P* = 0.054). **(C)** No statistical difference was found for row 1 or row 2 average stereociliary widths per bundle between Di-4 (18 hair bundles, 3 cochleae, 3 animals) and FM 4-64FX conditions (14 hair bundles, 2 cochleae, 2 animals) (row 1: *P* = 0.52, row 2: *P* = 0.077). Horizontal lines indicate comparisons using the Mann-Whitney U test: ns *P* > 0.05.

We next determined the stereociliary widths of live-stained IHCs in the two litters of slightly different age. In each 3-D reconstructed hair bundle, we created a virtual section through the bundle below the beveled portion of row 2 (Figure 2A). We determined a stereocilium’s width by measuring the shortest line passing through a stereocilium’s axis with endpoints on its perimeter’s midsection (see section “Materials and Methods,” Supplementary Figure 4, and Supplementary Movies 4, 5). When comparing the two litters using the live-staining conditions, we found no statistical difference between the widths of row 1 stereocilia (Di4: 0.45 ± 0.04 μm, FM 4-64FX: 0.45 ± 0.04 μm, *P* = 0.23) or between those of row 2 stereocilia (Di4: 0.45 ± 0.04 μm, FM 4-64FX: 0.47 ± 0.05 μm, *P* = 0.054) (Figure 2B and Supplementary Figure 5A). Similarly, we found no statistical difference between the average stereociliary widths per bundle of row 1 (Di4: 0.45 ± 0.03 μm, FM 4-64FX: 0.45 ± 0.03 μm, *P* = 0.52) or row 2 between conditions (Di4: 0.46 ± 0.02 μm, FM 4-64FX: 0.47 ± 0.03 μm, *P* = 0.077) (Figure 2C and Supplementary Figure 5B). Although the heights changed greatly within 1 day, the widths did not.

### Row 1 and 2 Stereociliary Dimensions Are Drastically Reduced After Scanning-Electron-Microscopy-Sample Preparation

**FIGURE 3:**
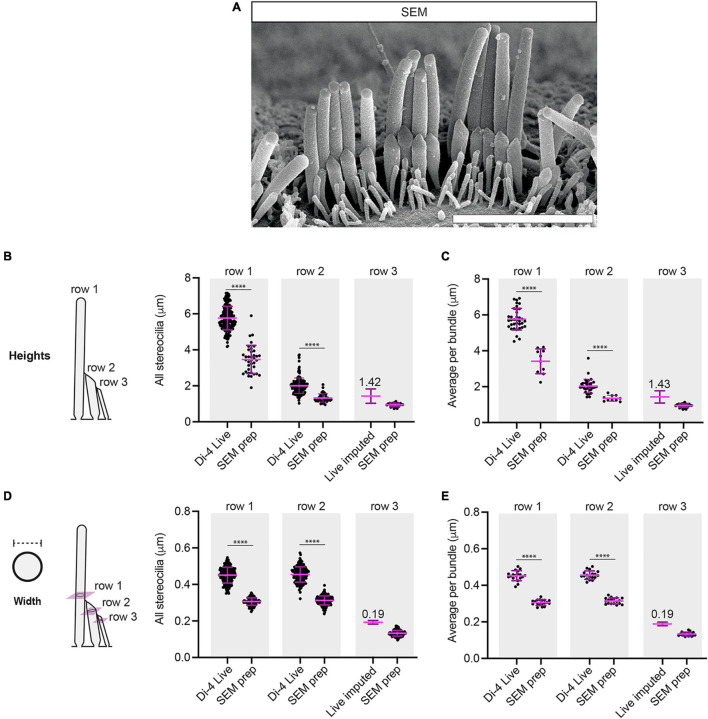
SEM preparation greatly reduces the heights and the widths of IHC stereocilia. **(A)** A representative image is shown of a P11 IHC hair bundle used for stereociliary height and width measurements. Scale bar: 3 μm. **(B)** SEM preparation greatly reduces row 1 and row 2 stereociliary heights compared to Di-4 live conditions (SEM: row 1: 37 stereocilia, row 2: 42 stereocilia, row 3: 36 stereocilia, 2 cochleae, 2 animals). **(C)** SEM preparation greatly reduces row 1 and row 2 average stereociliary heights per bundle (SEM: 10 hair bundles, 2 cochleae, 2 animals). **(D)** SEM preparation greatly reduces row 1 and row 2 stereociliary widths compared to Di-4 live conditions (SEM: row 1: 119 stereocilia, row 2: 136 stereocilia, row 3: 116 stereocilia, 2 cochleae, 2 animals). **(E)** SEM preparation greatly reduces row 1 and row 2 average stereociliary widths per bundle (SEM: 18 hair bundles, 2 cochleae, 2 animals). In all panels, row 3 live heights and widths were imputed from the measured SEM row 3 means and SDs, using row 2 means and SDs to determine the scaling factors between SEM and live measurements. Horizontal lines indicate comparisons using the Mann–Whitney *U*-test: ^****^P < 0.0001.

We next compared the live dimensions to dimensions obtained with electron microscopy. We chose conventional SEM (over TEM or Focused Ion Beam-SEM) because it allowed us to take several different measurements from each hair bundle and to repeat those measurements across many hair bundles. However, conventional SEM involves a harsh sample-preparation process: the tissue is fixed with paraformaldehyde and glutaraldehyde and subsequently subjected to dehydration. Understanding the extent to which stereociliary dimensions are affected by the SEM preparation process is of paramount importance, as it has been and still is widely used to determine stereociliary dimensions, which inform models of hair-bundle mechanics (Zetes, 1995; Furness et al., 1997; Smith and Chadwick, 2011; Ó Maoiléidigh and Hudspeth, 2013; Nam et al., 2015). Because stereocilia in SEM images are nearly always at an angle relative to the image plane, the absolute heights of stereocilia cannot be measured directly from individual 2-D images (Figure 3A). Therefore, to calculate the heights of stereocilia, we used paired images of the same bundle taken at two different angles (images were related by a eucentric rotation centered at the base of row 2, in the middle of the hair bundle), and used geometry to determine the heights from vectors in 3-D space (see section “Materials and Methods”). For stereocilia with visible apical-surface insertion sites, we used the full height measurements of the stereocilia from each image (Supplementary Figure 6A). However, for row 1 stereocilia with obscured insertion sites, we selected those that were paired with a row 2 stereocilium with a visible insertion site (Supplementary Figure 6B). We then measured the angles and heights of the visible portion of each row 1 stereocilium (from the tip of the row 2 stereocilium to the tip of the row 1 stereocilium) in both images. By using the angles and heights of the row 2 stereocilium and additionally calculating the angle of the apical surface of the hair cell relative to one of the image planes, we could determine the intersection point of the row 1 stereocilium in the apical surface and calculate the full height of the stereocilium. SEM height measurements are accurate only if a stereocilium is straight and close to vertical within an SEM image (Material and Methods). Because stereocilia at the edge of a row were rarely vertical in SEM images, we measured centrally located stereocilia.

For comparison with the Di-4 live-staining experiment, samples were prepared for SEM from littermates. When comparing the heights obtained from Di-4 live-staining to those calculated from SEM, we found that the stereociliary heights from SEM-samples were greatly reduced both for row 1 and for row 2 (SEM: row 1: 3.46 ± 0.78 μm, percentage difference = −40 ± 18% relative to Di-4; row 2: 1.32 ± 0.23 μm, percentage difference = −34 ± 25% relative to Di-4, *P* < 0.0001 for both) (Figure 3B). A similar large reduction was found when comparing the average height per bundle for both row 1 and row 2 (SEM: row 1: 3.42 ± 0.69 μm, percentage difference = −41 ± 16% relative to Di-4; row 2: 1.35 ± 0.16 μm, percentage difference = −34 ± 22% relative to Di-4, *P* < 0.0001 for both) (Figure 3C). While neither the live-lipophilic dye nor the phalloidin were clear enough to obtain row 3 stereociliary measurements, SEM allowed us to determine row 3 heights (0.94 ± 0.10 μm for individual stereocilia; 0.95 ± 0.07 μm for the hair-bundle average). Using these measurements and SEM-shrinkage factors obtained from row 2 Di-4 and SEM heights, we imputed live row 3 heights (1.42 ± 0.42 μm for all stereocilia; 1.43 ± 0.34 μm for the hair-bundle average) (Figures 3B,C).

We also compared width measurements from the Di-4 live staining and the SEM images. Widths for SEM were measured perpendicular to the long axes of fully visible stereocilia and differ negligibly between paired images at different angles. For row 1, widths were taken at a height just above the row 2 tips. For row 2, widths were measured at the widest point across each stereocilium below its tip. We found that row 1 stereociliary widths were greatly reduced after SEM processing compared to Di-4 live-staining, as were row 2 stereociliary widths (SEM: row 1: 0.31 ± 0.02 μm, percentage difference = −32 ± 11% relative to Di-4; row 2: 0.31 ± 0.02 μm, percentage difference = −31 ± 11% relative to Di-4, *P* < 0.0001 for both) (Figure 3D). Similar reductions were found for average widths per hair bundle (SEM: row 1: 0.31 ± 0.02 μm, percentage difference = −32 ± 7% relative to Di-4; row 2: 0.31 ± 0.02 μm, percentage difference = −31 ± 7% relative to Di-4, *P* < 0.0001 for both) (Figure 3E). As for the height, we were able to determine row 3 widths from SEM (0.13 ± 0.02 μm for all stereocilia; 0.13 ± 0.01 μm for the hair-bundle average) and impute the live widths using SEM-shrinkage factors obtained from row 2 Di-4 and SEM widths (0.19 ± 0.03 μm for all stereocilia; 0.19 ± 0.02 μm for the hair-bundle average) (Figures 3D,E).

Taken together, these data show that SEM-preparation greatly reduces the dimensions of stereocilia in comparison to live-stained preparations and, by extension, to mildly-fixed preparations. Furthermore, our multi-condition analysis allows us to impute row 3 stereociliary dimensions that cannot be resolved in live or mildly-fixed phalloidin conditions.

### Separations Between Stereociliary Insertions in the Apical Surface Are Drastically Reduced After Scanning-Electron-Microscopy-Sample Preparation

**FIGURE 4:**
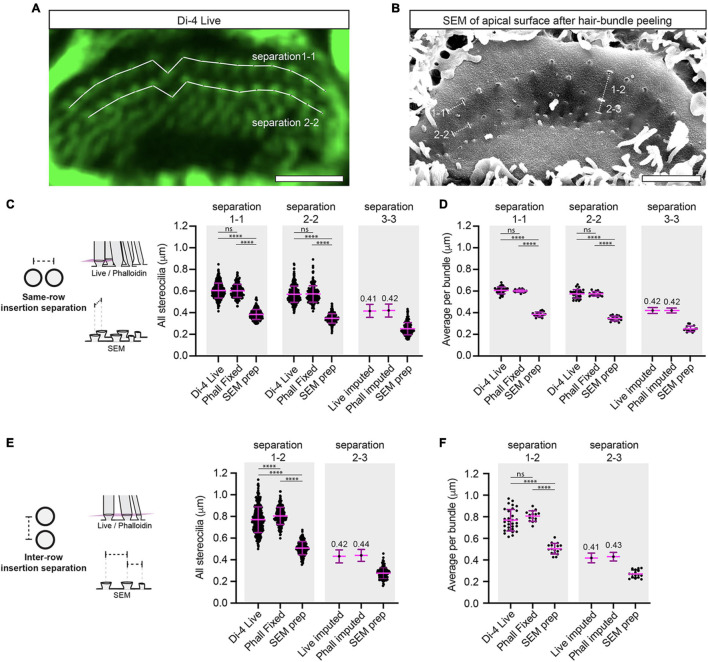
SEM preparation greatly reduces the separations between stereociliary insertion points. **(A)** A virtual section is shown through a 3-D reconstructed Di-4 live-stained hair bundle. The optical sectioning plane is parallel to and just above the apical surface and reveals the stereociliary insertion points. Stereociliary insertion separations were measured between the centers of the Di-4 spots. Scale bar: 3 μm. **(B)** A representative SEM image is shown of a hair cell’s apical surface with the hair-bundle peeled away using tape. Scale bar: 3 μm. **(C)** The insertion separation between stereocilia within the same row is greatly reduced in SEM compared to Di-4 and phalloidin-stained samples (separation 1-1: SEM: 205 measurements, Di-4: 360 measurements, phalloidin-stained: 191 measurements; separation 2-2: SEM: 180 measurements, Di-4: 348 measurements, phalloidin-stained: 183 measurements; separation 3-3: SEM: 175 measurements; 3 cochleae, 3 animals). Di-4 and phalloidin insertion separations are not statistically different (separation 1-1: *P* = 0.72, separation 2-2: *P* = 0.77). Row 3-3 separations for the Di-4 live and phalloidin-stained condition were imputed from the measured SEM row 3-3 mean and SD using row 2-2 means and SDs to determine scaling factors. **(D)** The average insertion separation per bundle between stereocilia of the same row is greatly reduced in SEM compared to Di-4 and phalloidin-stained samples (separation 1-1: SEM: 17 hair bundles, Di-4: 31 hair bundles, phalloidin-stained: 15 hair bundles; separation 2-2: SEM: 17 hair bundles, Di-4: 31 hair bundles, phalloidin-stained: 15 hair bundles; separation 3-3: 16 hair bundles, 3 cochleae, 3 animals). Di-4 and phalloidin average insertion separations per bundle are not statistically different (separation 1-1: *P* = 0.50, separation 1-1: *P* = 0.90). The row 3-3 insertion separations for the Di-4 live and the phalloidin-stained conditions were imputed from the measured SEM row 3-3 mean and SD using row 2-2 means and SDs to determine scaling factors. **(E)** The insertion separation between stereocilia of different rows is greatly reduced in SEM compared to the live and phalloidin-stained samples (separation 1-2: SEM: 189 measurements, Di-4: 369 measurements, phalloidin-stained: 196 measurements; separation 2-3: 154 measurements, 3 cochleae, 3 animals). Di-4 and phalloidin insertion separations between rows 1 and 2 are statistically different (separation 1-2: *P* < 0.0001). **(F)** The average insertion separation between stereocilia of different rows per bundle is greatly reduced in SEM compared to the live and phalloidin-stained samples (separation 1-2: SEM: 17 hair bundles, Di-4: 31 hair bundles, phalloidin-stained: 15 hair bundles; separation 2-3: SEM: 17 hair bundles; 3 cochleae, 3 animals). Di-4 and phalloidin average insertion separations between rows 1 and 2 per bundle are not statistically different (separation 1-2: *P* = 0.087). Horizontal lines indicate comparisons using the Mann-Whitney U test: ns *P* > 0.05, ^****^P < 0.0001.

Beyond the dimensions of individual stereocilia, we wanted to know whether SEM preparation affects the positions of stereocilia relative to each other. To assess this, we focused on the stereociliary insertion points in the apical surface of the hair cells. For live Di-4 stained hair bundles and the corresponding phalloidin-stained samples, we generated virtual sections above the cuticular plate (Figure 4A and Supplementary Movie 6). To measure the separations between the insertion points by SEM, we developed a procedure to peel away the hair bundles of SEM-samples after sample mounting, using tape, which reveals the insertion points (see section “Materials and Methods”) (Figure 4B). This method allowed us to image and measure stereocilia in an SEM sample and then image and measure insertions points in the same sample.

First, we measured and compared insertion separations within the same row. Compared to Di-4 staining, the SEM preparation showed reduced separations within row 1 and within row 2 (separation 1-1: 0.60 ± 0.07 μm for Di-4, 0.38 ± 0.05 μm for SEM, percentage difference = −37 ± 14% relative to Di-4; separation 2-2: 0.57 ± 0.07 μm for Di-4, 0.35 ± 0.04 μm for SEM, percentage difference = −39 ± 16% relative to Di-4, *P* < 0.0001 for both) (Figure 4C). This reduction was seen also when comparing hair-bundle averages within row 1 and row 2 (separation 1-1: 0.61 ± 0.03 μm for Di-4, 0.38 ± 0.02 μm for SEM, percentage difference = −37 ± 7% relative to Di-4; separation 2-2: 0.57 ± 0.04 μm for Di-4, 0.35 ± 0.02 μm for SEM, percentage difference = −40 ± 8% relative to Di-4, *P* < 0.0001 for both) (Figure 4D). We determined the insertion separations within row 3 using SEM (0.25 ± 0.06 μm for all stereocilia; 0.25 ± 0.03 μm for the hair-bundle average) and using the measured Di-4 and SEM row 2-2 separations, we imputed row 3-3 Di-4 insertion separations (0.41 ± 0.12 μm for all stereocilia; 0.42 ± 0.06 μm for the hair-bundle average) (Figures 4C,D).

Second, we compared insertion separations between stereocilia of different rows. We found that the row 1 to row 2 insertion separation was greatly reduced by SEM preparation compared to the Di-4 condition (separation 1-2: 0.77 ± 0.12 μm for Di-4, 0.51 ± 0.06 μm for SEM, percentage difference = −34 ± 18% relative to Di-4, *P* < 0.0001) (Figure 4E). Again, this observation was seen in the hair-bundle averages (separation 1-2: 0.77 ± 0.10 μm for Di-4, 0.50 ± 0.05 μm for SEM, percentage difference = −34 ± 15% relative to Di-4, *P* < 0.0001) (Figure 4F). We could determine the row 2 to row 3 insertion separation by SEM (0.28 ± 0.05 μm for all stereocilia; 0.27 ± 0.04 μm for the hair-bundle average) and impute its Di-4 value (0.42 ± 0.11 μm for all stereocilia; 0.41 ± 0.09 μm for the hair-bundle average) from the measured Di-4 and SEM row 1-2 insertion separations (Figures 4E,F).

Third, we compared the insertion separations obtained from live staining to those from mildly-fixed phalloidin samples. All insertion separation measurements were statistically indistinguishable between the two conditions, with the exception of the row 1-2 separation, but this percentage difference was small (all stereocilia: separation 1-1: *P* = 0.72, separation 2-2: *P* = 0.77, separation 1-2: *P* < 0.0001, see Table 1 for measurements, separation 1-2 for Di-4: 0.77 ± 0.12 μm, separation 1-2 for mildly-fixed: 0.80 ± 0.08 μm, separation 1-2 percentage difference = 4 ± 19% relative to Di-4; per bundle: separation 1-1: *P* = 0.50, separation 2-2: *P* = 0.90, separation 1-2: *P* = 0.087, see Table 2 for measurements) (Figures 4C–F).

**TABLE 1:** Heights, widths, and insertion separations under Di-4 live, mildly-fixed phalloidin, and SEM-prepared conditions determined from all stereocilia.

	Row	Live (Di-4)	Light fixation (Phalloidin)		SEM	
		Mean ± SD	Mean ± SD	% of Live	Mean ± SD	% of Live
	1	5.76 ± 0.65 μm (*n* = 195)	5.56 ± 0.35 μm (*n* = 219)	96 ± 13	3.46 ± 0.78 μm (*n* = 37)	60 ± 15
Height	2	2.00 ± 0.43 μm (*n* = 165)	1.95 ± 0.38 μm (*n* = 240)	98 ± 28	1.32 ± 0.23 μm (*n* = 42)	66 ± 18
	3	1.42 ± 0.42 μm (imputed from row 2)	1.39 ± 0.39 μm (imputed from row 2)	ND	0.94 ± 0.10 μm (*n* = 36)	ND

	1	0.45 ± 0.04 μm (*n* = 172)	0.37 ± 0.05 μm (*n* = 210)	82 ± 13	0.31 ± 0.02 μm (*n* = 119)	68 ± 8
Width	2	0.45 ± 0.04 μm (*n* = 149)	0.36 ± 0.04 μm (*n* = 196)	80 ± 12	0.31 ± 0.02 μm (*n* = 136)	69 ± 8
	3	0.19 ± 0.03 μm (imputed from row 2)	0.15 ± 0.03 μm (imputed from row 2)	ND	0.13 ± 0.02 μm (*n* = 116)	ND

Same row	1-1	0.60 ± 0.07 μm (*n* = 360)	0.60 ± 0.06 μm (*n* = 191)	100 ± 15	0.38 ± 0.05 μm (*n* = 205)	63 ± 11
insertion	2-2	0.57 ± 0.07 μm (*n* = 348)	0.57 ± 0.08 μm (*n* = 183)	100 ± 19	0.35 ± 0.04 μm (*n* = 180)	61 ± 11
separation	3-3	0.41 ± 0.12 μm (imputed from separation 2-2)	0.42 ± 0.12 μm (imputed from separation 2-2)	ND	0.25 ± 0.06 μm (*n* = 175)	ND

Inter row	1-2	0.77 ± 0.12 μm (*n* = 369)	0.80 ± 0.08 μm (*n* = 196)	104 ± 19	0.51 ± 0.06 μm (*n* = 189)	66 ± 13
insertion separation	2-3	0.42 ± 0.11 μm (imputed from separation 1-2)	0.44 ± 0.10 μm (imputed from separation 1-2)	ND	0.28 ± 0.05 μm (*n* = 154)	ND

*The number of stereocilia is indicated. ND, not determined.*

**TABLE 2:** Heights, widths, and insertion separations under Di-4 live, mildly-fixed phalloidin, and SEM-prepared conditions determined from hair-bundle averages.

	Row	Live (Di-4)	Light fixation (Phalloidin)		SEM	
		Mean ± SD	Mean ± SD	% of Live	Mean ± SD	% of Live
	1	5.76 ± 0.60 μm (*n* = 36)	5.57 ± 0.24 μm (*n* = 26)	97 ± 11	3.42 ± 0.69 μm (*n* = 10)	59 ± 13
Height	2	2.03 ± 0.39 μm (*n* = 36)	1.95 ± 0.22 μm (*n* = 26)	96 ± 21	1.35 ± 0.16 μm (*n* = 10)	66 ± 15
	3	1.43 ± 0.34 μm (imputed from row 2)	1.37 ± 0.25 μm (imputed from row 2)	ND	0.95 ± 0.07 μm (*n* = 10)	ND

	1	0.45 ± 0.03 μm (*n* = 18)	0.37 ± 0.02 μm (*n* = 18)	82 ± 7	0.31 ± 0.02 μm (*n* = 18)	68 ± 5
Width	2	0.46 ± 0.02 μm (*n* = 18)	0.37 ± 0.03 μm (*n* = 18)	80 ± 8	0.31 ± 0.02 μm (*n* = 18)	69 ± 5
	3	0.19 ± 0.02 μm (imputed from row 2)	0.16 ± 0.02 μm (imputed from row 2)	ND	0.13 ± 0.010 μm (*n* = 18)	ND

Same row	1-1	0.61 ± 0.03 μm (*n* = 31)	0.60 ± 0.01 μm (*n* = 15)	99 ± 6	0.38 ± 0.02 μm (*n* = 17)	63 ± 5
insertion	2-2	0.57 ± 0.04 μm (*n* = 31)	0.57 ± 0.02 μm (*n* = 15)	100 ± 8	0.35 ± 0.02 μm (*n* = 17)	60 ± 6
separation	3-3	0.42 ± 0.06 μm (imputed from separation 2-2)	0.42 ± 0.05 μm (imputed from separation 2-2)	ND	0.25 ± 0.03 μm (*n* = 16)	ND

Inter row	1-2	0.77 ± 0.10 μm (*n* = 31)	0.81 ± 0.05 μm (*n* = 15)	105 ± 15	0.50 ± 0.05 μm (*n* = 17)	66 ± 11
insertion separation	2-3	0.41 ± 0.09 μm (imputed from separation 1-2)	0.43 ± 0.08 μm (imputed from separation 1-2)	ND	0.27 ± 0.04 μm (*n* = 16)	ND

	1	ND	ND	ND	16.09 ± 1.51 (*n* = 11)	ND
Number of	2	ND	ND	ND	16.00 ± 2.36 (*n* = 10)	ND
stereocilia	3	ND	ND	ND	19.40 ± 0.89 (*n* = 5)	ND

Wing angle	1	ND	131°; 95% CI [127°, 135°] (*n* = 13)	ND	ND	ND
	2	ND	133°; 95% CI [128°, 139°] (*n* = 13)	ND	ND	ND

*The number of hair bundles is indicated. ND: not determined.*

In summary, we found that SEM preparation drastically reduces not only stereociliary heights and widths, but also the separation between stereociliary insertion points. Furthermore, we could use our SEM preparation to determine insertion separations that were not clear in live conditions, and impute the corresponding live-cell values.

### Apical Inner-Hair-Cell P11 Stereocilia Are Arranged in Two Wings Separated by a Notch

**FIGURE 5:**
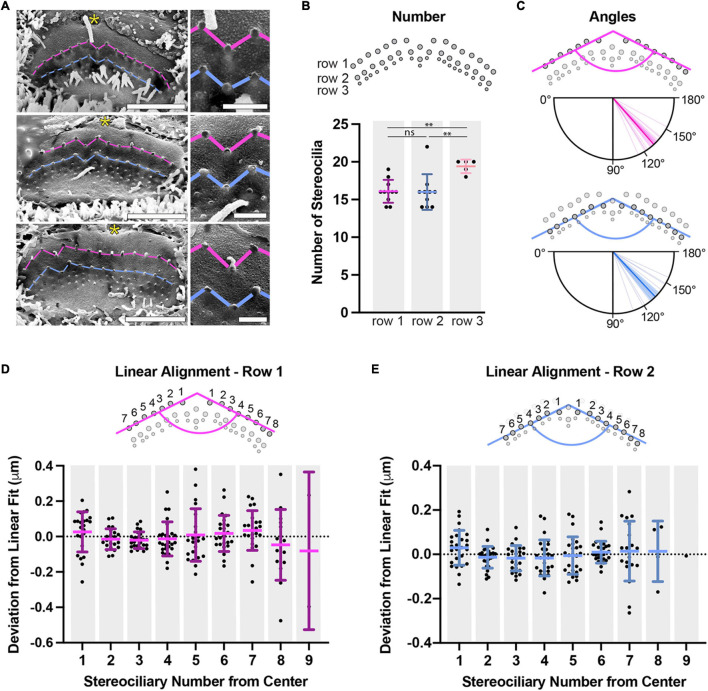
Apical IHC P11 stereocilia are arranged in two wings separated by a notch. **(A)** SEM images are shown of peeled IHC apical surfaces. The insertion points are connected by pink lines for row 1 and blue lines for row 2. IHC hair bundles form two wings separated by a central column that is shifted toward row 3, making a notch in the hair bundle. The fonticulus, the former kinocilium insertion point in the apical cell surface, is indicated by a yellow asterisk. Right panels show the notch at higher magnification. Scale bars: full view 2 μm, higher magnification 0.5 μm. **(B)** Stereociliary number per hair cell is shown, determined for each row from peeled hair-bundle SEM pictures (row 1 and 2: 11 hair bundles, row 3: 5 hair bundles). The number of row 1 stereocilia is the same as the number of row 2 stereocilia (*P* = 0.59), but the number of row 3 stereocilia is greater than that of row 1 or 2 (row 3 to 1: *P* = 0.0011, row 3 to 2: *P* = 0.010). **(C)** Hair-bundle angles were measured by identifying all row 1 or row 2 insertion point coordinates within a bundle, fitting each wing to a straight line, and using the line slopes (*n* = 13 hair bundles). Individual measurements are represented by thin lines, means are represented by thick lines, and 95% confidence intervals are represented by transparent colored sectors. **(D)** To determine if row 1 insertion points have additional structure beyond following a straight line, each stereocilium’s deviation from a line fit to its wing was determined. Stereocilia were labeled by a number (1–9) increasing from the central column to the edge of each wing. Note that the central column was not included. None of the average deviations for any stereociliary position were statistically different from zero (One sample *T*-test at the 95% confidence level against a mean of 0, Benjamini-Hochberg analysis), implying that there is no additional structure common to all bundles (*n* = 13). **(E)** The approach described in panel D was also applied to row 2 stereocilia (1–8) (*n* = 13). Horizontal lines indicate comparisons using the Mann-Whitney U test: ns *P* > 0.05, ^∗∗^*P* < 0.01.

Our peeled hair-bundle preparation gave us the opportunity to compare the insertion-point positions from many IHCs from the same cochlear location and look for patterns. We noticed that row 1 and 2 stereocilia were always arranged in two wings separated by a central column of stereocilia that was shifted toward row 3, forming an indentation or notch in the hair-bundle (Figure 5A). The notch was most commonly at the center of the bundle, but on rare occasions could be found at an eccentric position (3/24 cases), and in a single case the notch was formed by two columns of shifted stereocilia (data not shown). The notch is likely related to the former insertion position of the kinocilium in the apical surface, the fonticulus (Figure 5A; Kikkawa et al., 2008). The average number of stereocilia per row was similar between row 1 and row 2 (row 1: 16.1 ± 1.5; row 2: 16.0 ± 2.4; *P* = 0.59), but was higher for row 3 (19.4 ± 0.9; *P* < 0.01 for both comparisons) indicating that at this age, there are instances of multiple row 3 stereocilia connecting to a single row 2 stereocilium (Figures 3A, 5B).

To understand the layout of the insertion points further, we quantified and analyzed insertion points from mildly-fixed phalloidin images rather than the shrunken SEM preparations. The insertion-point coordinates of each hair-bundle wing were extracted and plotted. We found that the insertion positions within a wing are well-described by a straight line (row 1, *R*^2^ = 0.85 ± 0.12; row 2, *R*^2^ = 0.85 ± 0.15). Using the slope of each wing (determined from their linear fits), we calculated the hair-bundle angle for each row. There was a preferred angle for each row (*P* < 0.0001, Rayleigh z test). There was no statistical difference between the mean angle of 131° (95% CI [127°, 135°]) for row 1 and the mean angle of 133° (95% CI [128°, 139°]) for row 2 (*P* = 0.69, circular Mann Whitney U test) (Figure 5C). Stereocilia from vestibular end-organs and OHCs have been described as being positioned on a hexagonal or pseudohexagonal grid (Engström and Engström, 1978; Pickles et al., 1984; Comis et al., 1985; Bagger-Sjöbäck and Takumida, 1988; Roth and Bruns, 1992; Tsuprun and Santi, 1998, 2002; Rowe and Peterson, 2004; Beurg et al., 2006; Jacobo and Hudspeth, 2014). We tested whether P11 apical IHC stereocilia were positioned on a hexagonal grid, which implies an angle of 120° between their wings. Both row 1 and row 2 mean angles were statistically larger than 120° refuting the hypothesis (120° was outside the von Mises 95% confidence intervals). Furthermore, the hair-bundle shape could not be described as flat, as the angle was statistically smaller than 180° (180° was outside the von Mises 95% confidence intervals). Finally, we asked whether the stereociliary positions within a wing had additional structure by calculating the deviation of each insertion point from the linear fit line (Figures 5D,E). Stereocilia were numbered from 1 to 9 according to their position relative to the central column. None of the means for any of the insertion-position deviations in either row were statistically different from zero (one sample *T*-test against mean 0, Benjamini-Hochberg analysis), implying the absence of additional systematic structure along the hair-bundle wings. The systematic deviation from an angle of 120° and the systematically different separations within rows and between rows imply that IHC stereociliary insertion points lie on a pseudohexagonal grid and that this divergence from a hexagonal grid is not caused by biological variability.

### The Dimensions and Separations of Live Stereocilia

**FIGURE 6:**
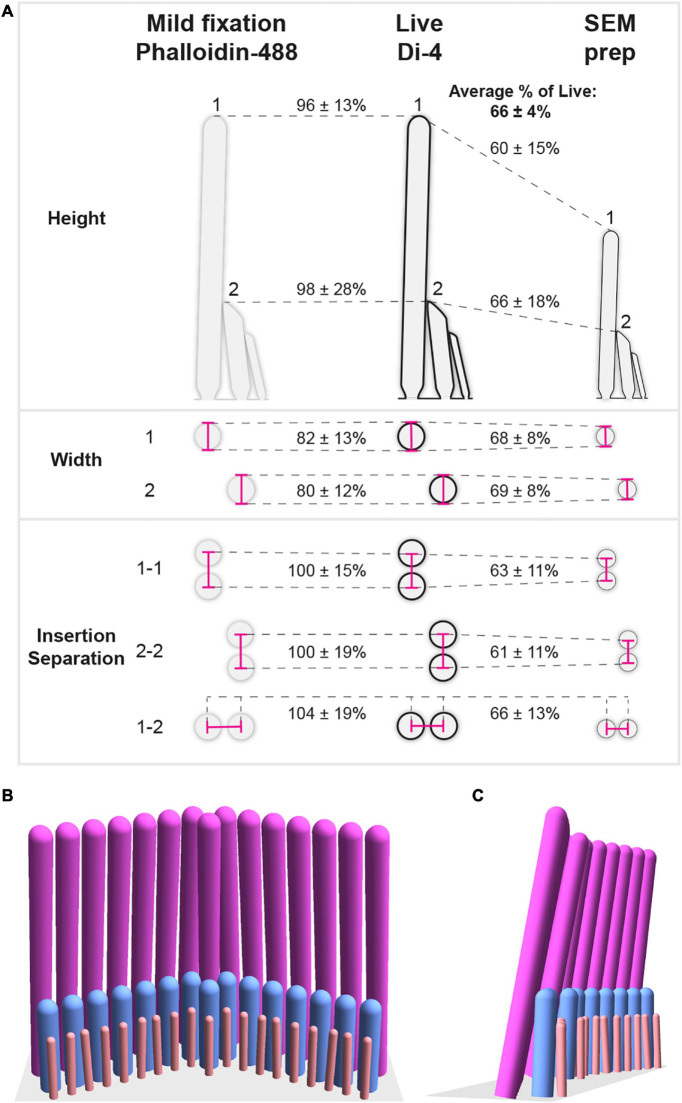
Scaling factors between live and mildly-fixed or SEM-prepared conditions and a 3-D model of a P11 apical living IHC hair bundle. **(A)** The illustration summarizes the morphological differences between live (Di-4), mildly-fixed (phalloidin), and SEM-prepared hair bundles. Mean percentages of live hair-bundle dimensions (heights, widths, and insertion separations) are indicated (± SDs). **(B)** A three-dimensional model is shown of a P11 apical living IHC hair bundle based on mean measurements (Table 1) and the mean angles and stereociliary numbers (Table 2). The angle of row 3’s wings has been chosen to be equal to row 2’s angle, creating a range of row 2-3 insertion separations (401–489 nm) consistent with measurements (Figure 4E). Stereocilia are inclined toward each other as follows: First, row 1 stereocilia are inclined 12° from the vertical toward row 2, in agreement with previous measurements (Furness et al., 1997), and inclined toward each other to create minimum gap of 50 nm between neighboring stereocilia; Second, row 2 stereocilia are inclined toward row 1 stereocilia to create minimum gaps of 20 nm between row 1-2 pairs; Third, each row 3 stereocilium is inclined toward the row 2 stereocilium with the closest insertion point to create minimum gaps of 20 nm between row 2-3 pairs. Since there are more stereocilia in row 3 than in row 2, some row 2 stereocilia are paired with two row 3 stereocilia.

When comparing all of our datasets, we find remarkable consistency in the changes (or lack thereof) that different preparation methods incur (Figure 6A). We find that live-stained and mildly-fixed samples have similar heights for both row 1 and row 2, while SEM preparation reduces the height of row 1 (SEM/Live% = 60 ± 15%) and row 2 (SEM/Live% = 66 ± 18%). Similarly, SEM widths are consistently reduced compared with live values (SEM/Live% = 68 ± 8% for row 1, SEM/Live% = 69 ± 8% for row 2). As expected, the stereociliary actin-core width (imaged with phalloidin-488) was smaller than the stereociliary membrane width (row 1 width Phall/Live% = 82 ± 13%, row 2 width Phall/Live% = 80 ± 12%) (Supplementary Figure 7), but the percentage difference was highly uncertain. Furthermore, insertion separations are reduced in SEM samples compared to live and mildly-fixed samples for row 1-1 separations (SEM/Live% = 63 ± 11%), row 2-2 separations (SEM/Live% = 61 ± 11%), and row 1-2 separations (SEM/Live% = 66 ± 13%). Calculating the shrinkage factors between different preparations enabled us to determine the live heights, widths, and separations for row 3 stereocilia. The individual measurements (Table 1) and averages per bundle (Table 2) define the morphology of a living hair bundle with the greatest accuracy and precision to date. Combining the individual measurements, we generated a 3-D representation of an apical IHC hair bundle (Figure 6B), which mirrors the 3-D reconstructions from live samples (Figures 1D,H), further validating the consistency of the measurements.

### Live-Cell Measurements Determine a Hair Bundle’s Mechanical Properties

We took advantage of our multidimensional datasets to determine some of the mechanical properties of the living hair bundle and to test whether these properties are affected by SEM shrinkage. The response of a hair bundle to stimulation is controlled by many factors, including its stiffness, the fluid coupling between stereocilia, and the relationship between channel gating and stereociliary deflection (Corey and Hudspeth, 1983; Ó Maoiléidigh and Hudspeth, 2013; Fettiplace, 2017). We have determined these three factors for an apical P11 IHC.

**FIGURE 7:**
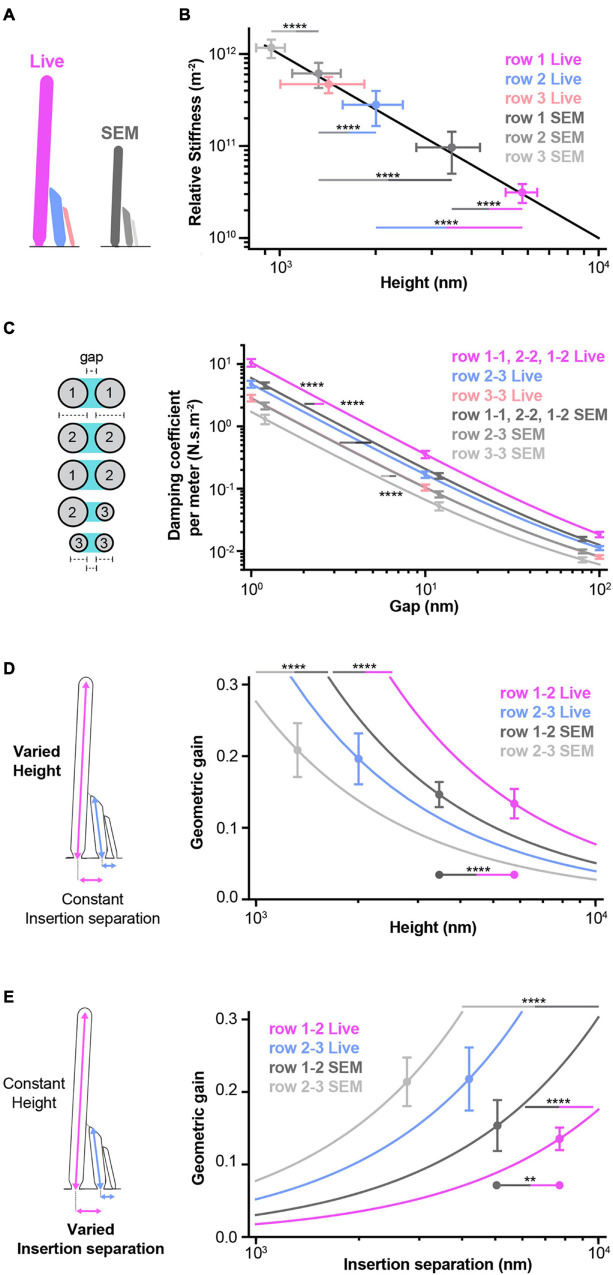
Models predict how morphology affects hair-bundle mechanics. **(A)** A schematic summarizes the measurements that were used to generate the results shown in this figure. Live-cell and SEM predictions are, respectively shown in color (pink, blue, and light pink) or shades of gray, according to row identity. All live-cell heights, widths, and separations in this figure correspond to Di-4 measurements. **(B)** A stereocilium’s deflection stiffness relative to its pivot stiffness is shown as a function of its height (black line, Eq. 1). The deflection stiffnesses are shown for stereocilia in each row based on live-cell and SEM heights (dots). For row 3, the live-cell height is imputed from the SEM height using a scaling factor based on row 2 live-cell and SEM heights. The live row 1 deflection stiffness is smaller than that of row 2 (asterisks, blue-pink line). Row 1 and 2 live deflection stiffnesses are smaller than those of SEM (asterisks, gray-pink and gray-blue lines). The SEM row 1 deflection stiffness is smaller than that of row 2, which is smaller than that of row 3 (asterisks, gray lines). **(C)** Fluid-coupling strength (damping coefficient per meter) between two stereocilia is shown as a function of the gap between pairs of stereocilia (Eq. 2). Owing to their similar widths, fluid coupling within and between stereocilia in rows 1 and 2 is similar and is combined into a single group (live-cell widths: 0.45 ± 0.04 μm for row 1, 0.45 ± 0.04 μm for row 2, percentage difference = 1 ± 13% relative to row 1; SEM widths: 0.31 ± 0.02 μm for row 1, 0.31 ± 0.02 μm for row 2, percentage difference = 2 ± 11% relative to row 1). For row 3, the live-cell width is imputed from the SEM width using a scaling factor based on row 1 and 2 live-cell and SEM widths. At any gap size, live row 1-1, 2-2, and 1-2 fluid coupling is larger than that of SEM (asterisks, gray-pink line). At any gap size, SEM row 1-1, 2-2, and 1-2 fluid coupling is larger than that of row 2-3, which is larger than that of row 3-3 (asterisks, gray lines). **(D)** The geometric gain between two stereocilia is shown as a function of the taller stereocilium’s height (Eq. 3). At any height, the live row 1-2 geometric gain is larger than that of SEM (asterisks, gray-pink line). At any height, the SEM row 1-2 geometric gain is larger than that of row 2-3 (asterisks, gray line). Geometric gains are shown at the means of measured heights (dots) (row 1-2: live-cell γ_12_ = 0.13 ± 0.02, SEM γ_12_ = 0.15 ± 0.02, percentage difference = –10 ± 20% relative to live-cell; row 2-3: live-cell γ_23_ = 0.20 ± 0.04, SEM γ_23_ = 0.21 ± 0.04, percentage difference = –6 ± 26% relative to live-cell). At the means of the measured heights, the row 1-2 live and SEM geometric gains are statistically different, but the percentage difference is highly uncertain (asterisks, gray-pink line between dots). **(E)** The geometric gain is shown as a function of the insertion separation (Eq. 3). At any insertion separation, the live row 1-2 geometric gain is smaller than that of SEM (asterisks, gray-pink line). At any insertion separation, the SEM row 1-2 geometric gain is smaller than that of row 2-3 (gray line). Geometric gains are shown at the means of measured separations (dots) (row 1-2: live-cell 0.14 ± 0.02, SEM 0.15 ± 0.04, percentage difference = –14 ± 28 relative to live-cell; row 2-3: live-cell 0.22 ± 0.04, SEM 0.21 ± 0.03, percentage difference = 2 ± 25% relative to live-cell). At the means of the measured insertion separations, the row 1-2 live and SEM geometric gains are statistically different, but the percentage difference is highly uncertain (asterisks, gray-pink line between dots). **(D,E)** For row 2-3, the live-cell insertion separation is imputed from the SEM separation using a scaling factor based on row 1-2 live-cell and SEM separations. Each dot and error bar represents the mean ± SD. Horizontal lines indicate comparisons using the Mann-Whitney U test: **P < 0.01, ****P < 0.0001. Statistical comparisons with live row 3 values cannot be performed, because these values are imputed from row 1, row 2, and SEM values.

The stiffness of an IHC bundle is dictated by the stiffnesses of its stereocilia – which are in turn determined by their heights and pivot stiffnesses – and the links between stereocilia. However, we have limited information about these components. Live stereociliary height measurements allow us to determine the extent to which stereociliary stiffness differs between rows owing to differences in stereociliary heights. Stereocilia pivot at their insertion point into the hair-cell’s apical surface (Howard and Ashmore, 1986; Crawford et al., 1989; Karavitaki and Corey, 2010; Wang et al., 2021; Figure 7A). A stereocilium’s deflection stiffness K_d_ relative to its pivot stiffness K_p_ is given by


(1) $$\frac{{\text{K}}_{d}}{{\text{K}}_{p}}=\frac{1}{{\text{H}}^{2}},$$

in which H is the height of the stereocilium. Whether there are differences in pivot stiffness between rows is not known, but the deflection stiffness relative to the pivot stiffness quantifies the extent to which stereociliary height affects the deflection stiffness. The deflection stiffness of a stereocilium increases rapidly when its height decreases (Figure 7B). Due to their live-cell height differences, row 1 stiffness is smaller (11 ± 5% of row 2) than that of row 2, which is smaller (60 ± 27% of row 3) than that of row 3. Owing to SEM-sample shrinkage, SEM stiffnesses are larger (219 ± 112 to 308 ± 165%) than the corresponding live-cell stiffnesses.

Pairs of neighboring stereocilia are strongly coupled by the fluid between them (Zetes, 1995; Zetes and Steele, 1997; Baumgart, 2011; Kozlov et al., 2011). Live-cell width measurements allow us to determine the extent to which this coupling depends on the gap between stereocilia and the extent to which coupling varies across a bundle. At a given height above the apical surface, the fluid-coupling force per unit length equals the relative velocity of the stereociliary pair times a damping coefficient per unit length λ, which depends on the gap between the stereocilia (Figure 7C). This damping coefficient quantifies the fluid-coupling strength and depends on the minimum gap g_m_ between and the widths of the stereociliary pair w_a_ and w_b_ at a given height above the apical surface according to (see section “Materials and Methods”):


(2a) $$\lambda =\frac{4\mu \pi }{\alpha -\beta -\tanh\left(\alpha -\beta \right)},$$

in which μ is the viscosity of water and


(2b) $$\alpha =\mathrm{arcosh}\left(1+\frac{g_{\text{m}\;}w_{\text{b}\;}}{w_{\text{a}\;}\left(w_{\text{a}\;}+w_{\text{b}\;}\right)}\right)\mathrm{and}$$

$$\beta =-\mathrm{arcosh}\left(1+\frac{g_{\text{m}\;}w_{\text{a}\;}}{w_{\text{b}\;}\left(w_{a}+w_{\text{b}\;}\right)}\right).$$

The minimum gap g_m_ decreases with distance from the apical surface, causing the fluid-coupling to increase rapidly with distance from the apical surface. Because live-cell row 1 and 2 stereocilia widths were comparable to each other and wider than row 3 stereocilia, fluid coupling within and between rows 1 and 2 is larger (166 ± 21 to 221 ± 42% as g_m_ decreases) than that between rows 2 and 3, which is in turn larger (139 ± 14 to 167 ± 30% as g_m_ decreases) than that within row 3. Due to SEM-sample shrinkage, SEM fluid coupling is smaller (57 ± 10 to 76 ± 8% of live) than the corresponding live-cell fluid coupling.

Live-cell measurements of stereociliary heights and insertion separations allow us to determine the relationship between gating-spring extensions and stereociliary deflections. The more a gating-spring extends the greater the probability of mechanotransduction-channel opening (Corey and Hudspeth, 1983; Howard and Hudspeth, 1988; Ó Maoiléidigh and Ricci, 2019). The gating-spring extension between a pair of stereocilia is proportional to the deflection of the taller stereocilium with a constant of proportionality, known as the geometric gain γ, given by


(3) $$\gamma =\frac{\text{s}}{{\text{H}}_{\text{t}\;}},$$

in which s is the separation between the pair of insertion points and H_t_ is the height of the taller stereocilium (Howard and Hudspeth, 1988; Jacobs and Hudspeth, 1990; Geisler, 1993; Pickles, 1993; Furness et al., 1997). The geometric gain decreases with the taller stereocilium’s increasing height (Figure 7D). At a given height, the live-cell row 1-2 geometric gain is larger (184 ± 56% of row 2-3) than the row 2-3 gain, and the SEM gains are smaller (row 1-2: 66 ± 13% of live, row 2-3: 66 ± 21% of live) than their corresponding live-cell gains. At the means of the measured heights, however, the live-cell geometric gains differ little from the corresponding SEM gains (SEM/Live% = 110 ± 21% for row 1-2, SEM/Live% = 106 ± 27% for row 2-3), because SEM-shrinkage is similar for heights and insertion separations (SEM/Live% = 60 ± 15% for row 1 height, SEM/Live% = 66 ± 18% for row 2 height, and SEM/Live% = 66 ± 13% for row 1-2 insertion separation).

The geometric gain increases with insertion separation (Eq. 3, Figure 7E). At a given separation, the live-cell row 1-2 gain is smaller (34 ± 8% of row 2-3) than the row 2-3 gain and the SEM gains are larger (row 1-2: 172 ± 44% of live, row 2-3: 149 ± 38% of live) than the corresponding live-cell gains. At the means of the measured separations, however, the live-cell geometric gains differ little from the corresponding SEM gains (SEM/Live% = 113 ± 36% for row 1-2, SEM/Live% = 104 ± 38% for row 2-3), because SEM-shrinkage is similar for heights and insertion separations.

## Discussion

The hair bundle, the mechanosensory organelle of hair cells, is central to our sense of hearing and its pathology. Yet, its live dimensions remained uncertain, which has limited our understanding of the hair bundle’s response to mechanical stimulation. Here, we rigorously measured or imputed the live stereociliary heights, widths, and insertion separations of C57BL/6J mouse P11 cochlear apical IHCs. Parallel sample processing, imaging, and comparisons showed that: 1) SEM preparation results in a hair bundle at a 1:1.5 scale compared to the live preparation while still preserving bundle proportions, which allows SEM dimensions to be converted into live dimensions; and, 2) in contrast to SEM, mildly-fixed/phalloidin-labeled samples have stereociliary heights, widths, and insertion separations similar to those from live conditions, validating mild fixation as a proxy for the living condition. Overall, we have generated the first comprehensive blueprint of a living hair bundle. Finally, we used our blueprint to calculate hair-bundle mechanical properties and showed that SEM measurements lead to the overestimation of stereociliary stiffness and underestimation of the fluid coupling between stereocilia, but accurately estimate the relationship between gating-spring extension and stereociliary deflection due to conservation of proportions. Thus, this study demonstrates the importance of using live hair-bundle dimensions to faithfully investigate hair-bundle function.

### Hair-Bundle Structure

The hair bundle develops at the apical surface of hair cells from the late embryonic stage until adulthood, morphing from a group of brush-border like microvilli to a stereociliary staircase of specific dimensions (Tilney et al., 1980; Tilney and DeRosier, 1986; Roth and Bruns, 1992; Kaltenbach et al., 1994; Waguespack et al., 2007; Krey et al., 2020). The final stereociliary dimensions and arrangement depends on the animal species, sensory organ, position within an organ, and hair-cell type (Garfinkle and Saunders, 1983; Wright, 1984; Kaltenbach et al., 1994; Zine and Romand, 1996; Ricci et al., 1997; Xue and Peterson, 2006; Xiong et al., 2012; Yarin et al., 2014). These hair-bundle variations indicate biological specialization of the structure for particular mechanical stimuli. This structure is still not fully understood at either the mechanical or developmental levels. Obtaining dimensions of unlabeled and live stereocilia is challenging due to their micron-scale heights and nano-scale widths. For these reasons, the vast majority of the reported stereociliary dimensions correspond to either transmission or scanning EM (Lim, 1980; Garfinkle and Saunders, 1983; Pickles et al., 1984; Roth and Bruns, 1992; Holme et al., 2002; Tsuprun et al., 2003; Furness et al., 2008; Zampini et al., 2011; Xiong et al., 2012; Taylor et al., 2015; Tarchini et al., 2016; Hadi et al., 2020) or to fixed, permeabilized, and actin-core labeled experiments (Krey et al., 2020). Although it was previously known that EM sample preparation shrinks cochlear tissue, the extent of this shrinkage at the stereociliary level had not been well quantified, and the consequences of phalloidin-staining preparation remained undetermined (Tilney et al., 1980; Wright, 1981; Forge et al., 1992). Another layer of uncertainty surrounding the previously reported stereociliary dimensions is that most have not been allocated to a well-defined position along the cochlear axis, or well-controlled for age. As we have shown here, controlling for age is critical: even a slight natural delay in development between litters of the same age had a large impact on the stereociliary heights. We minimized biological and experimental variability by limiting our analysis to a defined portion of the apical turn and by comparing cochleae from the same animals treated differentially. We found that the heights of row 1 and row 2 stereocilia were similar when imaged in live or mildly-fixed samples. Much in the same way as larger cochlear dimensions are unaffected by paraformaldehyde fixation (Edge et al., 1998), the micron-scale stereocilia appear to be unaffected, and mild fixation can be used as a proxy for live conditions. SEM sample preparation shrunk all measured dimensions and distances to the same degree (SEM/Live% = 66 ± 3 % on average) relative to the live conditions (Figure 6 and Tables 1, 2). We used scale factors to convert the SEM measurements of the small row 3 stereocilia to live values. Across all measurements, the average scaling factor between SEM and live measurements was 1:1.51 ± 0.08. In this particular apical cochlear location at P11, in live conditions, IHC row 1 is on average 2.9 times taller than row 2 and 4 times taller than row 3. Row 2 is 1.4 times taller than row 3. The width of rows 1 and 2 are similar and 2.3–2.4 times larger than row 3 widths. Our row 1 and 2 results are consistent with the ones obtained from phalloidin staining of a slightly more basal segment of the mouse cochlea (Krey et al., 2020). In particular, Krey et al. showed that after P7.5 the width of row 1 increases while the width of row 2 decreases, and at P11 that row 1 and row 2 transiently have the same width, as we have observed. The SEM scaling factor we determined can be used for other types of hair bundles as long as their SEM preparation and their SEM dimensions are similar to those reported here. The consequences of other EM sample preparation procedures on stereocilia dimensions would have to be determined; for example, sample preparations for TEM are expected to affect dimensions less than SEM preparation (Nordestgaard and Rostgaard, 1985).

In this study, we have also determined the arrangement of the stereocilia relative to each other in these P11 apical IHCs. To observe the stereociliary insertions, previous studies have used sonication or paper blotting to remove the stereocilia from samples during SEM sample preparation (Tilney and Saunders, 1983; Peterson et al., 1996; Severinsen et al., 2003). In this work, to sequentially image the stereocilia and the insertions of the same sample, we have developed a simple alternative method, using tape to peel-off the stereocilia from mounted SEM-samples. This method gives the added benefit of allowing mounted hair bundles to first be imaged, then peeled off, and finally re-imaged to collect insertion data, thereby allowing measurement of a wide spectrum of dimensions from a single sample. Stereociliary insertion patterns of cochlear hair cells have been reported for bird and turtle (Tilney and Saunders, 1983; Hackney et al., 1993), rodent OHCs (Gulley and Reese, 1977; Comis et al., 1985; Forge et al., 1988, 1991; Roth and Bruns, 1992; Souter et al., 1995; Tsuprun and Santi, 1998; Tsuprun et al., 2003), but rarely for rodent IHCs (Gulley and Reese, 1977; Forge et al., 1988; Furness et al., 2008). Our hair-bundle peeling method allowed us to image by SEM the insertion pattern of a substantial number of cells from the same cochlear location (*n* = 24). We observed consistently that row 1 and row 2 stereocilia have insertion positions with a central column shifted forward, creating a notch in the hair bundle. When imaging the hair bundle with intact stereocilia using SEM, the IHC insertion pattern is not evident, which explains why the stereociliary arrangements of rows 1 and 2 in IHCs have been described as forming an almost straight line (Engström and Engström, 1978; Forge et al., 1988; Ciganović et al., 2017). Two reports describe a “W” shape caused by the notch in adult chinchilla and guinea pig, although the insertion positions are not visible (Lim, 1986; Furness and Hackney, 2006). In contrast, a “W” shape caused by the notch in OHCs has been described many times (Lim, 1986; Forge et al., 1988; Roth and Bruns, 1992; Tsuprun et al., 2003; Furness et al., 2008). The notch is likely related to the kinocilium. At P11, the IHC kinocilium is either absent or degenerating, but its former insertion position in the apical surface, the fonticulus, remains and is visible by SEM (Kikkawa et al., 2008). It will be important to further investigate when the notch appears during hair bundle development, if it is maintained at later ages, and finally, if it is seen along the tonotopical axis.

When investigating the shape of the bundle, we found that both the row 1 and row 2 wings of the IHCs form the same angle, that the angle differs from 120 and 180°, and that insertion points along the wings do not diverge systematically from a straight line. Furthermore, insertion separations are not uniform across all rows, with row 1-2 separation being larger than both row 1-1 and row 2-2, all of which are larger than row 2-3 and row 3-3 separations. These observations imply that the IHC insertion positions deviate systematically from a hexagonal array.

Each stereocilium’s orientation is defined by two leaning angles, a polar and an azimuthal angle. We did not quantify these angles, as we expect our sample preparations to alter the stereociliary angles from their *in-vivo* states. For example, in Di-4 and phalloidin images, we often see row 1 stereocilia pointing away from each other, splitting the bundle (Figure 1). In most cases, SEM preparation causes clear disorganization in the leaning angles (Figure 3A). Although the polar angle has previously been measured from TEM images, these measurements also indicate that sample preparation changed the angle (Zetes, 1995; Furness et al., 1997). For some measurements, row 1 leans away from row 2, which is inconsistent with a force balance at rest between insertion-point pivots and links that causes the bundle to move forward when tip links are cut (Tobin et al., 2019). Determining the *in-vivo* leaning angles remains a challenge for future work.

### Hair-Bundle Function

Our understanding of hair-bundle mechanics is based on a combination of experimental data and modeling, which rely on accurate and precise measurements. We found that SEM measurements underestimate IHC bundle dimensions, resulting in greatly overestimated stereociliary stiffness (219 ± 112 to 308 ± 165%) and greatly underestimated fluid coupling (57 ± 10 to 76 ± 8% of live). In contrast, SEM provides good estimates of geometric gains, because heights and insertion separations shrink similar amounts. Similarly, the stiffness of row 1 relative to row 2 (live-cell: 11 ± 5%; SEM: 16 ± 9%) and row 2 relative to row 3 (live-cell: 60 ± 27%: SEM: 53 ± 20%) is well-estimated by SEM, because all heights shrink by similar amounts.

The stiffness of an individual stereocilium determines its deflection in response to stimulation (Corey et al., 2017; Fettiplace, 2017; Ó Maoiléidigh and Ricci, 2019). Here we show that the deflection stiffness of row 1 is 11 ± 5% and 7 ± 2% of rows 2 and 3, respectively, assuming similar pivot stiffnesses. A stiffness gradient within the hair bundle would affect gating of the mechanotransduction channel. However, rootlet differences between the rows might cause pivot-stiffness differences (Furness et al., 2008). How these specializations affect hair-bundle function remains to be determined.

In addition to the effects of stereociliary stiffness on bundle deflection, fluid coupling between stereocilia is very large and is thought to ensure coherent stereociliary motion across a bundle (Zetes, 1995; Zetes and Steele, 1997; Kozlov et al., 2011). This coupling increases rapidly as the gap between pairs of stereocilia decreases, but we lack accurate measurements of the gap size at the point of closest apposition. It is not possible to resolve the smallest gaps optically, but gaps can be resolved using electron microscopy. EM imaged gaps seen in IHCs and OHCs range from 1 to 100 nm, which may imply different amounts of fluid coupling across a bundle or might be a consequence of hair-bundle damage during sample preparation (Osborne et al., 1984; Pickles et al., 1984; Furness and Hackney, 1985; Santi and Anderson, 1987; Furness et al., 1997; Tsuprun et al., 2003; Zampini et al., 2011; Hackney and Furness, 2013). Here we show that fluid coupling at a given gap size within and between rows 1 and 2 is stronger than that between rows 2 and 3, which is again stronger than that within row 3, because stereocilia in rows 1 and 2 are wider than those in row 3.

Finally, gating of the mechanotransduction channel depends on several factors including stereociliary deflections, heights, and insertion separations. The most common formulation of the gating-spring model for mechanoelectrical transduction assumes that all gating springs extend the same amount in response to hair-bundle deflection, characterized by a single geometric gain for each hair cell (Corey and Hudspeth, 1983; Howard and Ashmore, 1986; Howard and Hudspeth, 1988; Crawford et al., 1989; Jacobs and Hudspeth, 1990; Karavitaki and Corey, 2010; Ó Maoiléidigh and Hudspeth, 2013; Corey et al., 2017; Fettiplace, 2017; Tobin et al., 2019; Ó Maoiléidigh and Ricci, 2019). Previous work using TEM indicated that the IHC row 1-2 gating spring extends twice as much as that of row 2-3 (average 167 ± 50%; characteristic frequency 0.27–13 kHz), but was not conclusive owing to measurement uncertainties (Furness et al., 1997). Indeed, only one-to-six stereociliary pairs at each cochlear position were observed, and it is not known whether TEM preparation shrinks hair-bundle dimensions equally or the extent to which it changes the polar leaning angles of the stereocilia – measurements which were used to calculate gating-spring extension relative to hair-bundle deflection (Zetes, 1995). We overcome these limitations by measuring over 150 stereocilia per dimension, by using live-cell, mildly-fixed, and SEM measurements, and by determining that SEM shrinkage is similar across dimensions. To avoid the uncertainty associated with the polar leaning angle, we calculate the geometric gain relative to the taller stereocilium of each pair without accounting for the polar leaning angle, because accounting for the polar leaning angle changes the geometric gain very little (<2% for a row 1 angle of 12° toward row 2) (Zetes, 1995; Furness et al., 1997). We find that the row 1-2 geometric gain (γ_12_ = 0.14 ± 0.03) is smaller (66 ± 22% of row 2-3) than that of row 2-3 (γ_23_ = 0.20 ± 0.06). The geometric gains allow us to determine if row 1-2 and 2-3 gating springs extend similar amounts in response to IHC bundle deflection. For a bundle deflection *X*, the row 1-2 gating spring extends by γ_12_*X* = (0.14 ± 0.03)*X*. To a good approximation, row 2 has the same angular displacement as row 1 and the row 2-3 gating spring then extends by γ_23_(H_2_/H_1_)*X* = (0.07 ± 0.03)*X*, in which H_1_ and H_2_ are, respectively the heights of rows 1 and 2. In agreement with previous work, the gating-springs do not extend the same amount: row 1-2 gating-spring extension is double that of row 2-3 (190 ± 79%) (Furness et al., 1997). For small stimuli, this difference will cause row 2 mechanotransduction channels to respond by changing their open probability twice as much as row 3’s. Additional experimentation and modeling will be required to understand the full consequences of these differences.

While previous work has provided measurements of IHC bundle morphology using electron and optical microscopy, it has been limited by uncertain cochlear locations, uncertain ages, differences in species, or artifacts in sample-preparation (Garfinkle and Saunders, 1983; Pickles et al., 1984; Holme et al., 2002; Tsuprun et al., 2003; Beurg et al., 2008; Furness et al., 2008; Zampini et al., 2011; Nam et al., 2015; Taylor et al., 2015; Tarchini et al., 2016; Liu et al., 2019; Tobin et al., 2019; Hadi et al., 2020; Krey et al., 2020). Here we control for these uncertainties and show definitively that apical mouse IHC bundles have several morphological specializations. Row 1 is much taller than rows 2 and 3. Stereocilia in rows 1 and 2 are much wider than in row 3. Row 1-2 insertion separations are larger than those within rows 1 and 2, which are larger than those of row 2-3 and those within row 3. Row 1 and 2 angles are larger than 120° and smaller than 180°. Our modeling demonstrates several conspicuous mechanical consequences of IHC bundle specializations, but the functional reasons for these specializations remain elusive.

## Conclusion

This work provides the first comprehensive dataset of live hair-bundle dimensions, which are of paramount importance for determining hair-bundle function. Furthermore, the SEM-to-live scaling factor we determined will be instrumental for generating live blueprints from rare samples, such as human hair bundles.

## Materials and Methods

### Animals

The Administrative Panel on Laboratory Animal Care (APLAC) at Stanford University (protocol #28278) approved all animal procedures. C56BL/6J adult mice were purchased from Jackson Laboratories (Bar Harbor, ME, United States) and bred to produce pups.

### Cochlear Tissue Preparation

Inner ears of P11 mice of both sexes were dissected from temporal bones at room temperature (RT) in extracellular recording solution containing the following: 145 mM NaCl, 2 mM KCl, 2 mM CaCl_2_, 1 mM MgCl_2_, 10 mM 4-(2-hydroxyethyl)-1-piperazineethanesulfonic acid (HEPES), 6 mM Glucose, 2 mM pyruvate, 2 mM ascorbic acid, and 2 mM creatine monohydrate. The pH of the external solution was adjusted to 7.4 by addition of NaOH and osmolality ranged from 304 to 308 mOsmol. The apical turn of the organ of Corti was gently dissected out of the cochlea and the tectorial membrane was removed. To minimize differences due to development, animals from within the same litters were compared. One ear of each animal was used for immediate live imaging, while the other was used for mildly-fixed conditions and imaged after the live one. As shrinkage was expected in SEM samples, cochleae from the littermates of the live and mildly-fixed animals were used for comparisons.

### Live Stereociliary-Membrane Staining

Dissected cochlear apical turns were transferred with a spoon to a dish containing a lipophilic dye and stained for 5 min at RT while protected from light, then transferred to a recording dish with external recording solution and held in place with dental floss ensuring that IHC hair bundles were oriented vertically for imaging. We used the lipophilic dye ANEP (aminonaphthylethenylpyridinium) dye Di-4-ANEPPDHQ (D36802, ThermoFisher Scientific) (MW: 666 g/mlole). The dye was resuspended in 100% ethanol at 1 mg/ml (1.3 mM), and then diluted at 15 μg/ml (19 μM final) in external recording solution before each staining. We also used the lipophilic styryl dye FM 4-64FX (F34653, Invitrogen) (MW: 788 g/mole), which produces low fluorescence in water and intense fluorescence upon binding to the plasma membrane. The dye was resuspended in water at 200 μg/ml (357 μM), and then diluted at 5 μg/ml (9 μM final) in external recording solution before each staining.

### Actin-Core Fluorescent Imaging of Mildly-Fixed Hair Bundles

Dissected cochlear apical turns were transferred with a spoon to a dish containing fixative (4% Paraformaldehyde (PFA) aqueous solution (RT15714, Electronic Microscopy Sciences) in 0.05 mM Hepes buffer pH 7.2, 10 mM CaCl_2_, 5 mM MgCl_2_, 0.9% NaCl) and incubated for 30 min at RT. The sample was then transferred to a new dish containing 0.5% Triton and phalloidin-Alexa 488 (A12379) (1/800) in external recording solution for 15 min, causing permeabilization and acting labeling. The sample was then transferred to a recording dish for imaging.

### Live Hair-Bundle Fluorescence Imaging

Live and mildly-fixed IHC hair bundles were imaged in external recording solution using a Zeiss LSM880 microscope in Airyscan mode and a Zeiss Plan Apochromat 40X water immersion 1NA lens and a X7 digital zoom. Di-4-ANEPPDHQ (in lipid, Excitation 472 nm, Emission 615 nm) and phalloidin-488 (Excitation 490 nm, Emission 525 nm) were excited with a 488 nm laser, yielding a theoretical maximum lateral resolution of 246 nm and 210 nm and an axial resolution of 1145 nm and 978 nm. FM 4-64FX (in lipid, Excitation 565 nm, Emission 744 nm) was excited with a 561 nm laser, yielding a theoretical maximum lateral resolution of 298 nm and an axial resolution of 1385 nm. Emission filters used were band-pass at 495–550 nm for phalloidin-488 and long-pass at 570 nm for Di-4-ANEPPDHQ and FM 4-64FX. To limit physical damage to the sample, the laser power used was ∼4.5% for live imaging, and 5–7% for phalloidin staining, in which the maximum laser power was approximately 5.5 μW for the 488 laser and 49.8 μW for the 561 laser. The microscope stage was controlled along the z-axis with a Heidenhain drive, which has a z-axis resolution of 0.05 μm and a z-axis repeatability of ± 0.1 μm. Image stacks of 330-nm thickness encompassing the entire hair bundles (15–20 optical sections per bundle) of 5–8 consecutive IHCs were deconvolved using ZEN software (blue edition, Zeiss).

An imaging system’s optical resolution is the smallest distance between two points at which they can be distinguished and depends on the system’s point-spread function. The resolution of our system is sufficient to measure row 1 and 2 heights, widths, and insertion separations. The resolution is also sufficient to measure row 2-3 and 3-3 insertion separations, but the fluorescence signal from row 3 was too dim relative to the background to precisely localize row 3 insertion positions. Using 100 nm diameter beads, we determined the point-spread function’s full width at half maximum (FWHM) to be 802 ± 25 nm in the axial direction and to be 201 ± 8 nm and 224 ± 12 nm in the lateral directions (488 nm laser, *n* = 5 for each measurement). For distinguishable objects, the optical resolution is larger than the precision in distance measurements (Thompson et al., 2002; von Diezmann et al., 2017). A wider point-spread function causes more variability in a distance measurement because it becomes increasingly difficult to place the measurement points at the desired positions within an image. This variability contributes to the distance standard deviation, which quantifies the precision of a distance measurement. The precision of a measurement is not limited to the width of the point-spread function, however, and can be more than an order of magnitude smaller than this width (Thompson et al., 2002). The standard deviations of our measurements are larger than this achievable limit, because we placed measurement points manually, we are not imaging isolated molecules, and our samples include biological variability. Across all stereociliary measurements based on Di-4 or phalloidin, the smallest lateral standard deviation of 40 nm is consistent with the lateral FWHMs and the smallest axial standard deviation of 350 nm is consistent with the axial FWHM. Averaging measurements per hair bundle reduces the standard deviations further.

### Stereociliary Height, Width, and Insertion-Separation Measurements From Fluorescence Imaging

Airyscan processed stacks were transferred to Imaris (Oxford Instruments, United States) software. The stacks were visualized using the 3-D View interface. The best volume renderings for measurements were obtained when the optical sections were perpendicular to the stereocilia. Stereociliary heights from row 1 and row 2 were measured by manually placing measurement points at the stereociliary base and top in 3-D space. We defined these points as the location at which the fluorescence signal suddenly decays. We determined stereociliary widths at the position just below the beveled portion of row 2. There, we generated a virtual section with the Imaris slicer tool. A stereocilium’s cross section looks like a distorted oval because a slice is usually oblique to the stereocilium’s axis and a stereocilium’s membrane cannot be distinguished from the membranes of neighboring stereocilia when they are closer than the imaging system’s optical resolution (Supplementary Figure 4). To minimize error caused by this distortion, we determined a stereocilium’s width by measuring the length of the shortest line in 3-D, with endpoints placed on the perimeter’s midsection, that passes through the center of the stereocilium’s ovate cross section. The shortest line in 3-D may not appear to be the shortest in 2-D and may not appear to lie on the midsection in 2-D (Figure 2A and Supplementary Figure 4).

Insertion separations were determined from phalloidin-stained hair bundles, from the first virtual section parallel to and above the hair cell’s apical surface. Separations were defined to be between stereociliary centers.

### Stereociliary Number Count

Using the SEM images of the apical surface of IHCs with the stereocilia peeled off, we identified stereociliary rows using the notch as a reference. Numbers of stereocilia were counted (including the central stereocilia) only for rows that were fully visible. Rows that were obstructed by remaining stereocilia or other objects were not included in the quantification.

### Hair-Bundle Angle Determination and Stereociliary Arrangement

From phalloidin insertion-point images, stereociliary xy-coordinates were extracted (WebPlotDigitizer), plotted, and fit to straight lines. The slope of each hair-bundle wing was determined from their linear fits, and the angle of the bundle was calculated from these slopes. Mean bundle angles were calculated using the vector components, and confidence intervals were determine using a von Mises 95% confidence-interval chart (Batschelet, 1981).

Each stereocilium was assigned a number (1–9) as its distance from the indented central column, with 1 being directly adjacent to the column. The perpendicular deviation of a stereocilium’s coordinates from the linear fit line was calculated using


(4a) $$r\frac{\left|b+{\mathrm{mx}}_{s}-y_{s}\right|}{\sqrt{1+m^{2}}},$$

in which (x_s_,y_s_) is the insertion point coordinate, m is the slope of the linear fit, b is the y intercept, and r determines the sign of the deviation, such that r is equal to 1 if


(4b) $$\left(y_{s}-\frac{m\left(x_{s}+{\mathrm{my}}_{s}-\mathrm{mb}\right)}{m^{2}+1}-b\right)>0$$

and r is equal to −1 if


(4c) $$\left(y_{s}-\frac{m\left(x_{s}+{\mathrm{my}}_{s}-\mathrm{mb}\right)}{m^{2}+1}-b\right)<0.$$

The average deviations were tested against a mean of zero at the 95% confidence level using one-sample *t*-tests and Benjamini-Hochberg analysis (false discovery rate 25%).

### Sample Preparation for Scanning Electronic Microscopy

Samples were prepared as previously described (Trouillet et al., 2021). Briefly, inner ears were isolated in external recording solution, transferred with a spoon to a dish containing fixative (4% Paraformaldehyde aqueous solution (RT15714, Electronic Microscopy Sciences) in 0.05 mM Hepes buffer pH 7.2, 10 mM CaCl_2_, 5 mM MgCl_2_, 0.9% NaCl) and incubated for 30 min at RT. The cochleae were then dissected in the fixative to remove the stria vascularis, Reissner’s membrane, and the tectorial membrane. The samples were re-fixed in 2.5% glutaraldehyde and 4% PFA in 0.05 mM HEPES Buffer pH 7.2, 10 mM CaCl_2_, 5 mM MgCl_2_, and 0.9% NaCl overnight at 4°C, then washed, dehydrated in ethanol (30%, 75%, 95%, 100%, and 100%, 5 min incubation) and brought to the critical drying point using Autosamdri-815A (Tousimis). Cochleae were mounted on 45° beveled-studs using silver paint. The front and back sides of hair bundles were coated with sequential 3 nm palladium depositions (sputter coater EMS150TS; Electron Microscopy Sciences and custom-made stud adaptor). Samples were imaged at 5 kV at a working distance of 4 mm on a FEI Magellan 400 XHR Field Emission Scanning Electron Microscope (Stanford Nano Shared Facilities) and its TLD secondary-electron detector in immersion mode. In this mode, the spatial resolution is 0.8 nm. To limit stereociliary damage and displacement during imaging, the beam current was frequently reduced from 50 to 25 pA or 13 pA. To ensure that beam was not displacing the sample, consecutively scanned images were compared. If a drift was observed, the corresponding hair bundle was not used further. Two stereo-pair images of the same IHC bundle at 20-25,000X were obtained differing by a 5-degree front/back eucentric rotation centered at the insertion point of a row 2 stereocilium located in the middle part of the hair bundle. The microscope was periodically calibrated for measurements using a SIRA-type calibration specimen for ultra-high-resolution modes with 2% or less error between 50 and 350 KX magnifications at our imaging settings.

### Determination of Stereociliary Widths and Heights From Scanning-Electron-Microscopy Pictures

Measured dimensions correspond to hair bundles coated on the back and front with 3 nm of palladium, which approximately equals 1.9% of the diameter of row 1 and 2 stereocilia and 4.6% of the diameter of row 3 stereocilia.

Widths for SEM were measured horizontally across the fully-visible parts of stereocilia. For row 1, widths were measured at a height just above the row-2 tips. For row 2, widths were measured at the widest point across the stereocilia below the tip bevel. For row 3, widths were measured at the widest point across the stereocilia below the tips. To be counted as a row 3 stereocilium, a stereocilium was required to be in front of row 2 in an SEM image and to abut a row 2 stereocilium.

The method we use to determine a stereocilium’s height from two tilted SEM images is a corrected version of a published method (Li et al., 2020). Between images, we rotate the sample counterclockwise by an angle α around an axis in the image plane, a eucentric rotation. We chose the rotation axis to be close to the stereocilium’s insertion point into the hair cell’s apical surface and define a coordinate system with an origin at the insertion point, an x-axis parallel to the rotation axis, and a y-axis in the image plane (Supplementary Figure 6A). Let the vectors **A** and **B**, respectively represent the three-dimensional height and positions of the stereocilium before and after the sample rotation and have coordinates {x_A_,y_A_,z_A_} and {x_B_,y_B_,z_B_}. The vectors **A**′ and **B**′ are the projections of the stereocilium onto the image plane and have coordinates {x_A_,y_A_,0} and {x_B_,y_B_,0}. Using each image, we measure the stereocilium’s projected heights |**A**′| and |**B**′| and its angles with the x-axis θ_A_ and θ_B_. The xy-coordinates of the stereocilium vectors are then given by


(5) $${\text{x}}_{A}=\left|{\text{A}}^{\prime }\right|\cos\theta_{A},$$

$${\text{y}}_{A}=\left|{\text{A}}^{\prime }\right|\sin\theta_{A},$$

$${\text{x}}_{B}=\left|{\text{B}}^{\prime }\right|\cos\theta_{B},\mathrm{and}$$

$${\text{y}}_{B}=\left|{\text{B}}^{\prime }\right|\sin\theta_{B}.$$

Assuming that the stereocilium is perpendicular to the x-axis, the angle between **A** and **B** is the rotation angle α. We find the height of the stereocilium H = |**A**| = |**B**| and the z-coordinates z_A_ and z_B_ using the law of cosines and the relationships between the height and the coordinates yielding


(6) $$H=\sqrt{\frac{\beta +\sqrt{\beta^{2}-4\left(1-\delta^{2}\right){\left(x_{A}y_{B}-x_{B}y_{A}\right)}^{2}}}{2\left(1-\delta^{2}\right)}},$$

$${\text{z}}_{\text{A}\;}=-\sigma \left(\alpha \right)\sigma \left({\text{y}}_{\text{B}\;}-{\text{y}}_{\text{A}\;}\right)\sqrt{{\text{H}}^{2}-\left|{\text{A}}^{\prime }\right|},\mathrm{and}$$

$${\text{z}}_{\text{B}\;}=\sigma \left(\alpha \right)\sigma \left({\text{y}}_{\text{A}\;}-{\text{y}}_{\text{B}\;}\right)\sqrt{{\text{H}}^{2}-\left|{\text{B}}^{\prime }\right|},$$

in which δ = *cos*⁡α, $\beta =x_{A}^{2}+x_{B}^{2}+y_{A}^{2}+y_{B}^{2}-2\delta x_{A}x_{B}-2\delta y_{A}y_{B}$, and


(7) $$\sigma \left(\text{x}\right)=\{\begin{array}{cc} 1,x\geq 0 & \\ -1,x<0 & \end{array}.$$

To find the height of a stereocilium whose insertion point is obscured by a shorter stereocilium **A**, we define the vector **C** from the tip of the shorter stereocilium to the tip of the obscured stereocilium (Supplementary Figure 6B). We define a coordinate system with an origin at the shorter stereocilium’s insertion point and measure |**A**′|, |**C**′|, θ_A_, and θ_C_. As described above, we find the coordinates of **A** {x_A_,y_A_,z_A_} and the coordinates of **C** relative to **A** {x_*CA*_,y_*CA*_,z_*CA*_}. The coordinates of the obscured stereocilium’s tip relative to the shorter stereocilium’s insertion point are then given by


(8) $${\text{x}}_{\text{C}\;}={\text{x}}_{\text{CA}\;}+{\text{x}}_{\text{A}\;},$$

$${\text{y}}_{\text{C}\;}={\text{y}}_{\text{CA}\;}+{\text{y}}_{\text{A}\;},\mathrm{and}$$

$${\text{z}}_{\text{C}\;}={\text{z}}_{\text{CA}\;}+{\text{z}}_{\text{A}\;}.$$

We assume that the apical surface plane of the hair cell is related to the first image plane by a counterclockwise rotation around the x-axis through the angle φ. To find φ, we chose two points M′ and N′ in the image plane that appear to be on the apical surface and that form a line perpendicular to the x-axis. We measure the projected length of the line |M′N′| and use the method described above to find the true length of the line |*MN*|. The angle φ is then given by


(9) $$\varphi =\frac{\pi }{2}+\arccos\left(\frac{\left|{\text{M}}^{{}^{\prime }}{\text{N}}^{{}^{\prime }}\right|}{\left|\text{MN}\right|}\right).$$

The coordinates of the obscured stereocilium’s insertion point Q are then given by


(10) $${\text{x}}_{\text{Q}\;}=\frac{{\text{x}}_{\text{C}\;}{\text{z}}_{\text{A}\;}-{\text{x}}_{\text{A}\;}{\text{z}}_{\text{C}\;}+\left({\text{x}}_{\text{A}\;}{\text{y}}_{\text{C}\;}-{\text{x}}_{\text{C}\;}{\text{y}}_{\text{A}\;}\right)\tan\text{φ}\;}{{\text{z}}_{\text{A}\;}-{\text{z}}_{\text{C}\;}+\left({\text{y}}_{\text{C}\;}-{\text{y}}_{\text{A}\;}\right)\tan\text{φ}\;},$$

$${\text{y}}_{\text{Q}\;}=\frac{{\text{y}}_{\text{C}\;}{\text{z}}_{\text{A}\;}-{\text{y}}_{\text{A}\;}{\text{z}}_{\text{C}\;}}{{\text{z}}_{\text{A}\;}-{\text{z}}_{\text{C}\;}+\left({\text{y}}_{\text{C}\;}-{\text{y}}_{\text{A}\;}\right)\tan\text{φ}\;},\mathrm{and}$$

$${\text{z}}_{\text{Q}\;}={\text{y}}_{\text{Q}\;}\tan\text{φ}\;.$$

The height of the obscured stereocilium H is finally given by


(11) $${\text{H}}^{2}={\left({\text{x}}_{\text{C}\;}-{\text{x}}_{\text{Q}\;}\right)}^{2}+{\left({\text{y}}_{\text{C}\;}-{\text{y}}_{\text{Q}\;}\right)}^{2}+{\left({\text{z}}_{\text{C}\;}-{\text{z}}_{\text{Q}\;}\right)}^{2}.$$

The method we use is based on several assumptions. For unobscured stereocilia, we assume that the rotation axis is in the image plane, the rotation axis is close to the insertion points, the x-axis is parallel to the rotation axis, and the stereocilia are approximately perpendicular to the x-axis. We make the same assumptions for obscured stereocilia along with the assumption that the surface plane is related to the image plane by a rotation around the x-axis. Using artificial images with stereocilia of known height, we estimate the error owing to these assumptions to be less than 10%.

### Hair-Bundle Peeling and Insertion-Separation Measurements

To measure the separation between stereocilia at the hair-cell’s apical surface, hair bundles from SEM-prepared samples were peeled-away using a piece of permanent double-sided tape (Scotch, 3M) placed on a fine forceps and gently applied to the hair-cell area. Samples were then reimaged and the number of stereocilia per row and the insertion separations were measured from top-down views. Stereocilia from different rows were paired starting from the most central row 1 stereocilium, which is shifted toward row 3, and then pairing the adjacent stereocilia. If multiple row 3 stereocilia could be paired to a single row 2 stereocilium, measurements were made using the closest stereocilium.

### Fluid Coupling Between Stereocilia

Because inertial fluid forces between stereocilia are negligibly small, we determine the fluid-coupling forces between two stereocilia by solving the corresponding inertialess Stokes equations (Baumgart, 2011). We calculate the fluid-coupling force per unit length between infinitely long parallel cylinders, because at a given height above the apical surface neighboring stereocilia are approximately parallel, have approximately circular cross sections, and their fluid coupling is dominated by forces distant from their ends. We first determine fluid coupling in the lubrication limit, when the gap between stereocilia is much smaller than their widths, by extending previous calculations of the fluid coupling between two cylinders of equal widths to the case in which their widths are not equal (Zetes, 1995; Zetes and Steele, 1997; Baumgart, 2011; Kozlov et al., 2011). In terms of an xy-coordinate system with an origin on the line connecting their centers, the distance g between them is given by


(12) $$\text{g}\left(y\right)=g_{\text{m}\;}+r_{\text{a}\;}+r_{\text{b}\;}-\sqrt{r_{\text{a}\;}^{2}-y^{2}}-\sqrt{r_{\text{b}\;}^{2}-y^{2},}$$

in which g_m_ is the minimum gap between the cylinders, and r_a_ and r_b_ are their radii (Supplementary Figure 8). When g_m_≪*min*⁡{r_a_,r_b_}, the distance g is well approximated by


(13) $$g\left(y\right)=g_{m}+\frac{r_{a}+r_{b}}{2r_{a}r_{b}}y^{2}.$$

When the cylinders come together or move apart along the line connecting their centers with relative velocity **U**, the Poiseuille flow created causes pressure given by


(14) p(y)=-12μ|U|∫-∞y′g(y′)3ydy′ =12μ|U|2ra3rb3(ra+rb)(2gmrarb+(ra+rb)y2)2,

in which μ is the viscosity of water (Zetes, 1995). The magnitude of the force per unit length owing to the pressure is then given by


(15) $$\left|{\text{F}}_{\mathrm{lub}}\right|=\int_{-\infty }^{\infty }pd\text{y}=3\sqrt{2}\pi \mu {\left(\frac{r_{a}r_{b}}{g_{m}\left(r_{a}+r_{b}\right)}\right)}^{\frac{3}{2}}\left|U\right|.$$

Note that w_a_ = 2r_a_ and w_b_ = 2r_b_ are the cylinder widths (Eq. 2). This calculation for a small gap helps us to determine the fluid coupling satisfying the Stokes equations for a gap of any size. To determine the fluid-coupling force for a gap of any size, we extend a previous calculation of the fluid coupling between two cylinders of equal widths to the case in which their widths are not equal (Wakiya, 1975). Following the notation of Wakiya, consider the two cylinders in bipolar coordinates {α,0} and {β,0}, and with minimum distances to the y-axis g_a_ and g_b_, such that the minimum gap between them is g_m_ = g_a_ + g_b_ (Supplementary Figure 8). Using Eqs. 2.5, 2.6, and 2.10 in Wakiya, we find the magnitude of the fluid coupling force between the cylinders is given by


(16) $$\left|{\text{F}}_{c}\right|=\frac{4\mu \pi \left|U\right|}{\alpha -\beta -\tanh\left(\alpha -\beta \right)}=\lambda \left|U\right|$$

in which α = *arcosh*(1 + g_a_/r_a_), β = −*arcosh*(1 + g_b_/r_b_), and λ is the damping coefficient per unit length. To complete the solution, we determine distances g_a_ and g_b_ that are consistent with the equations discussed by Wakiya. We find a constraint for g_a_ and g_b_ by matching the leading term of Eq. 16 expanded around g_a_ = 0 = g_b_ with the lubrication solution (Eq. 15), yielding


(17) $${\left(\sqrt{\frac{g_{\text{a}\;}}{r_{\text{a}\;}}}+\sqrt{\frac{g_{\text{b}\;}}{r_{\text{b}\;}}}\right)}^{2}=\frac{g_{\text{m}\;}\left(r_{\text{a}\;}+r_{\text{b}\;}\right)}{r_{\text{a}\;}r_{\text{b}\;}}.$$

We use this constraint and g_m_ = g_a_ + g_b_ to find


(18) $$g_{\text{a}\;}=\frac{g_{\text{m}\;}r_{\text{b}\;}}{r_{\text{a}\;}+r_{\text{b}\;}}\mathrm{and}g_{\text{b}\;}=\frac{g_{\text{m}\;}r_{\text{a}\;}}{r_{\text{a}\;}+r_{\text{b}\;}}.$$

Inserting these expressions into Eq. 16 yields the fluid coupling force for a gap of any size, in which


(19) $$\alpha =\mathrm{arcosh}\left(1+\frac{g_{\text{m}\;}r_{\text{b}\;}}{r_{\text{a}\;}\left(r_{\text{a}\;}+r_{\text{b}\;}\right)}\right)\mathrm{and}$$

$$\beta =-\mathrm{arcosh}\left(1+\frac{g_{\text{m}\;}r_{\text{a}\;}}{r_{b}\left(r_{a}+r_{\text{b}\;}\right)}\right).$$

The fluid-coupling force opposes the relative motion such that **F_c_** = −λ**U**. We only calculate the fluid-coupling force owing to motion along the line connecting the cylinders’ centers, because this force is much larger than fluid-coupling forces caused by other types of relative motion (Zetes, 1995; Zetes and Steele, 1997; Baumgart, 2011; Kozlov et al., 2011).

### 3-D Illustration of the Hair Bundle and Calculus

We used Mathematica 12.1.1.0 (Wolfram Research, Inc., Champaign, IL, United States) to create the 3-D hair-bundle model (Figures 6B,C), to calculate the effects of morphology on mechanics (Figure 7), and to create Supplementary Figures 4, 6.

### Statistical Analyses

Statistical tests performed were described in the text and calculated using Prism 9 (GraphPad Software, San Diego, CA, United States), Excel (Microsoft, Redmond, WA, United States), and Mathematica 12.1.1.0 (Wolfram Research, Inc., Champaign, IL, United States).

## Data Availability Statement

The raw data supporting the conclusions of this article will be made available by the authors upon request.

## Ethics Statement

The animal study was reviewed and approved by the Administrative Panel on Laboratory Animal Care (APLAC) at Stanford University (protocol #28278), which approved all animal procedures.

## Author Contributions

NG and DÓ conceived of the study. PA, KRM, and NG conducted the fluorescence imaging. NG conducted the SEM experiments. KKM, DÓ, and NG performed the analysis. NG and DÓ designed experiments. DÓ performed the mathematical modeling of the hair bundle, derived the fluid-coupling equations, and derived the SEM-height equations. NG, DÓ, and KKM wrote the manuscript. All authors contributed to the article and approved the submitted version.

## Conflict of Interest

The authors declare that the research was conducted in the absence of any commercial or financial relationships that could be construed as a potential conflict of interest.

## Publisher’s Note

All claims expressed in this article are solely those of the authors and do not necessarily represent those of their affiliated organizations, or those of the publisher, the editors and the reviewers. Any product that may be evaluated in this article, or claim that may be made by its manufacturer, is not guaranteed or endorsed by the publisher.

## Supplementary Material

Supplementary Figure 1 Stereociliary heights differ little between live-stained and mildly-fixed conditions. Points of the same color correspond to stereocilia of the same hair bundle, but color schemes differ between conditions and rows. Data is presented at a higher magnification below each plot. **(A)** Stereociliary heights for all stereocilia are shown from P11 WT IHCs in live Di-4 and mildly-fixed phalloidin conditions. Row 1 Di-4 and phalloidin heights are statistically different, but the percentage difference is small (*P* = 0.0027, percentage difference = −4 ± 13% relative to Di-4). Row 2 Di-4 and phalloidin heights are not statistically different (*P* = 0.22). **(B)** Stereociliary heights averaged per hair bundle are shown from P11 WT IHCs in live Di-4 and mildly-fixed phalloidin conditions. Row 1 Di-4 and phalloidin heights are not statistically different (*P* = 0.21). Row 2 Di-4 and phalloidin heights are not statistically different (*P* = 0.32). **(C)** Stereociliary heights for all stereocilia are shown from P11 WT IHCs in live FM 4-64FX and mildly-fixed phalloidin conditions. Row 1 FM 4-64FX and phalloidin heights are statistically different, but the percentage difference is small (*P* < 0.0001, percentage difference = −4 ± 13% relative to FM 4-64FX). Row 2 FM 4-64FX and phalloidin heights are statistically different, but the percentage difference is highly uncertain (*P* < 0.0001, percentage difference = 22 ± 33% relative to FM 4-64FX). **(D)** Stereocilia heights averaged per hair bundle are shown from P11 WT IHCs in live FM 4-64FX and mildly-fixed phalloidin conditions. Row 1 FM 4-64FX and phalloidin heights are statistically different, but the percentage difference is small (*P* = 0.017, percentage difference = −4 ± 7% relative to FM 4-64FX). Row 2 FM 4-64FX and phalloidin heights are statistically different, but the percentage difference is highly uncertain (*P* < 0.0001, percentage difference = 22 ± 19% relative to FM 4-64FX). Horizontal lines indicate comparisons using the Mann-Whitney U test: ns *P* > 0.05, ^∗^*P* < 0.05, ^∗∗^*P* < 0.01, ^∗∗∗^*P* < 0.001, ^****^*P* < 0.0001.

Supplementary Figure 2 Stereociliary height coefficient of variation per hair bundle and stereociliary height relative to row 1 stereociliary position. Coefficients of variation (SD/mean) are shown for row 1 **(A)** and row 2 **(B)** stereociliary heights from P11 WT IHCs live-stained with Di-4 or FM 4-64FX or mildly fixed and stained with phalloidin Alexa488 (36 hair bundles, 3 cochleae, 3 animals for Di-4; 25 hair bundles, 3 cochleae, 3 animals for phalloidin; 18 hair bundles, 2 cochleae, 2 animals for FM 4-64FX; 33 hair bundles, 2 cochleae, 2 animals for phalloidin). **(C)** Row 1 stereocilia are labeled with respect to the column forming the notch, which is at position 0 (Figure 5). An example of a phalloidin-Alexa488 mildly-fixed P11 IHC hair bundle (FM 4-64X) is labeled with the heights of each row 1 stereocilium. Note that the difference in height between the tallest and shortest row 1 stereocilium is more than 2 μm. Scale bars: 2 μm. **(D)** Row 1 stereociliary heights group by their position within the bundle are shown from phalloidin-Alexa488 mildly-fixed P11 IHCs (33 hair bundles, 2 cochleae, 2 animals) (Litter 2). The phalloidin heights of the position 0 and the position 1 stereocilia are statistically different, but the percentage differences are highly uncertain (stereociliary height: position 0: 4.40 ± 0.54 μm; position 1: 4.95 ± 0.41 μm; percentage difference = −11 ± 14% relative to position 1). **(E)** The heights of the position 1 and the last stereocilia within a hair bundle wing are compared. The comparison is based on measurements of phalloidin-Alexa488 mildly-fixed P11 IHCs (33 hair bundles, 2 cochleae, 2 animals) (Litter 2). The phalloidin heights of the position 1 and the last position stereocilia are statistically different, but the percentage differences are highly uncertain (stereociliary height: position 1: 4.95 ± 0.41 μm; last stereocilium: 4.01 ± 0.75 μm; percentage difference = −19 ± 17% relative to position 1). Horizontal lines indicate comparisons using the Mann-Whitney U test: ^****^*P* < 0.0001.

Supplementary Figure 3 Comparisons of stereociliary heights from phalloidin and live-stained conditions. **(A)** Row 1 but not row 2 stereociliary heights from P11 WT IHCs with phalloidin-labeling in two independent litters are different owing to developmental variability between litters, but the percentage difference is highly uncertain (*P* < 0.0001 for both row 1 and 2, row 1 percentage difference = −14 ± 10% relative to Litter 1, row 2 percentage difference = −8 ± 25% relative to Litter 1). **(B)** Average stereociliary height per hair bundle is shown from P11 WT IHCs with phalloidin-labeling in two independent litters. Row 1 and 2 heights are statistically different between the two litters (row 1: *P* < 0.0001, percentage difference = −14 ± 6% relative to Litter 1; row 2: *P* = 0.0056, difference = −8 ± 16% relative to Litter 1), but the row 2 percentage difference is too small and uncertain to be biologically important. **(C)** Stereociliary heights are shown from P11 WT IHCs with live staining in two independent litters (*P* < 0.0001 both row 1 and 2, row 1 percentage difference = −13 ± 15% relative to Litter 1, row 2 percentage difference = −27 ± 29% relative to Litter 1). Although the heights are statistically different, the percentage differences are highly uncertain. **(D)** Average stereociliary height per hair bundle is shown from P11 WT IHCs with live staining in two independent litters (*P* < 0.0001 both row 1 and 2, row 1 percentage difference −13 ± 11% relative to Litter 1, row 2 percentage difference = −27 ± 22% relative to Litter 1). Although the heights are statistically different, the percentage differences are highly uncertain. Magenta lines indicate means ± SDs. Horizontal lines indicate comparisons using the Mann-Whitney U test: ^∗∗^*P* < 0.01, ^****^*P* < 0.0001.

Supplementary Figure 4 The width of a cylindrical stereociliary section must be measured in 3-D. **(A)** A cylinder’s cross section is shown delimited by two planes perpendicular to a cylinder’s axis. **(B)** Viewed from the cylinder’s axis, a perpendicular cross section appears circular with a uniform perimeter thickness. The ends of a line (black) used to measure the cylinder’s width are placed on the perimeter’s midsection. The line is chosen to pass though the center of the cylinder’s cavity. **(C)** A cross section is shown delimited by two planes oblique to the cylinder’s axis. **(D)** Viewed from the cylinder’s axis, an oblique cross section appears oval with a nonuniform perimeter thickness. The ends of a line (black) used to measure the cylinder’s width are placed on the perimeter’s midsection. The line is chosen to be the shortest line that passes through the center of the cylinder’s cavity. **(B,D)** Light from planes perpendicular to the cylinder’s axis increase the apparent perimeter thickness in 2-D, which creates error in the determination of the cylinder’s width in 2-D.

Supplementary Figure 5 Stereociliary widths differ little between Di-4 and FM 4-64FX conditions. Points of the same color correspond to the same hair bundle, but row 1 and 2 color schemes differ between conditions and row. **(A)** Stereociliary widths are shown from P11 WT IHCs live-stained with Di-4 or FM 4-64FX. Data is presented at a higher magnification below each plot. Row 1 Di-4 and FM 4-64FX widths are not statistically different (*P* = 0.23). Row 2 Di-4 and FM 4-64FX widths are not statistically different (*P* = 0.054). **(B)** Stereociliary widths averaged per hair bundle are shown from P11 WT IHCs live-stained with Di-4 or FM 4-64FX. Row 1 Di-4 and FM 4-64FX widths are not statistically different (*P* = 0.52). Row 2 Di-4 and FM 4-64FX widths are not statistically different (*P* = 0.077). Horizontal lines indicate comparisons using the Mann-Whitney U test: ns *P* > 0.05.

Supplementary Figure 6 Schematics for SEM-height determination from paired images. **(A)** A stereocilium is represented by the vector **(A)** in a coordinate frame with an xy-plane coincident with an SEM-image plane. The projection **(A)′** onto the image plane is at an angle θ_A_ relative to the x-axis. Rotating the sample counterclockwise around the x-axis changes the coordinates such that the stereocilium is now represented by the vector **(B)**. The projection **(B)′** onto the image plane is at an angle θ_B_ relative to the x-axis. **(B)** A stereocilium, represented by **(A)**, has a projection **(A)′** that obscures the base of a taller stereocilium’s projection onto the image plane. The vector **(C)′** represents the visible part of the obscured stereocilium in an SEM image and is the projection of the vector **(C)** onto the image plane. The apical surface plane of the hair cell is related to the image plane by a counterclockwise rotation though the angle φ . The points N and M on the surface have projections N′ and M′ onto the image plane, such that the line through N′ and M′ is perpendicular to the x-axis. A line through **(C)** intersects the surface at the point Q . The distance from Q to the point C is the true height of the obscured stereocilium.

Supplementary Figure 7 Stereociliary width comparison between live, mildly-fixed phalloidin stained, and SEM-prepared samples. **(A)** A cartoon depicts stereociliary cross sections, upon which width measurements are based, corresponding to different sample-preparation methods. Virtual sections were taken through 3-D reconstructed fluorescently labeled hair bundles below row 2’s stereociliary tips. In Di-4 and FM 4-64FX live conditions, the staining labels the membrane, forming a ring. In phalloidin-488 mildly-fixed samples, the staining labels the actin core ensheathed by the stereociliary membrane, resulting in an filled-circle narrower than the membranous ring. In SEM preparation, the sample shrinks and the membrane is removed by the dehydration procedure. The dehydrated sample is coated with a thin layer of metal for imaging. **(B)** A representative virtual section trough a phalloidin-488 mildly-fixed hair bundle used for width measurements. Scale bar: 0.5 μm. **(C)** Stereociliary widths are shown from P11 WT IHCs live-stained with Di-4 (row 1: 172 stereocilia, row 2: 149 stereocilia, 3 cochleae, 3 animals), live-stained with FM 4-64FX (row 1: 101 stereocilia, row 2: 90 stereocilia, 2 cochleae, 2 animals), mildly-fixed with phalloidin (Di-4 litter: row 1: 210 stereocilia, row 2: 189 stereocilia, 3 cochleae, 3 animals) (FM 4-64FX litter: row 1: 318 stereocilia, row 2: 250 stereocilia, 2 cochleae, 2 animals), or prepared for SEM (Di-4 litter: row 1: 119 stereocilia, row 2: 136 stereocilia, 2 cochleae, 2 animals) (FM 4-64FX litter: row 1: 96 stereocilia, row 2: 100 stereocilia, 2 cochleae, 2 animals) (row 1: Di-4 vs. Phall: *P* < 0.0001, percentage difference = −18 ± 14% relative to Di-4; Di-4-paired Phall vs. SEM: *P* < 0.0001, percentage difference = −17 ± 14% relative to Phall; FM 4-64FX vs. Phall: *P* < 0.0001, percentage difference = −12 ± 15% relative to FM; FM-paired-Phall vs. SEM: *P* < 0.0001, percentage difference = −25 ± 15% relative to Phall; row 2: Di-4 vs. Phall: *P* < 0.0001, percentage difference = −20 ± 14% relative to Di-4; Di-4-paired-Phall vs. SEM: *P* < 0.0001, percentage difference = −14 ± 14% relative to Di-4; FM 4-64FX vs. Phall: *P* < 0.0001, percentage difference = −16 ± 15% relative to FM; FM-paired-Phall vs. SEM: *P* < 0.0001, percentage difference = 22 ± 15% relative to Phall). Although the widths are statistically different, most of the percentage differences are highly uncertain. **(D)** Averages of stereociliary widths per bundle are shown from P11 WT IHCs live-stained with Di-4 (rows 1 and 2: 18 hair bundle, 3 cochleae, 3 animals), live-stained with FM 4-64FX (rows 1 and 2: 14 hair bundles, 2 cochleae, 2 animals), mildly-fixed with phalloidin (Di-4 litter: rows 1 and 2: 18 hair bundles, 3 cochleae, 3 animals) (FM 4-64FX litter: rows 1 and 2: 26 hair bundles, 2 cochleae, 2 animals), or prepared for SEM (Di-4 litter. rows 1 and 2: 18 hair bundles, 2 cochleae, 2 animals), (FM 4-64FX litter. rows 1 and 2: 17 hair bundles, 2 cochleae, 2 animals) (row 1: Di-4 vs. Phall: *P* < 0.0001, percentage difference = −18 ± 8% relative to Di-4; Di-4-paired-Phall vs. SEM: *P* < 0.0001, percentage difference = −18 ± 8% relative to Phall; FM 4-64FX vs. Phall: *P* < 0.0001, percentage difference = −13 ± 10% relative to FM 4-64FX; FM 4-64FX -paired-Phall vs. SEM: *P* < 0.0001, percentage difference = −24 ± 9% relative to FM 4-64FX; row 2: Di-4 vs. Phall: *P* < 0.0001, percentage difference = −20 ± 9% relative to Di-4; Di-4-paired Phall vs. SEM: *P* < 0.0001, percentage difference = −14 ± 10% relative to Phall; FM 4-64FX vs. Phall: *P* < 0.0001, percentage difference = −16 ± 8% relative to FM 4-64FX; FM 4-64FX -paired-Phall vs. SEM: *P* < 0.0001, percentage difference = −21 ± 9% relative to Phall). Although the widths are statistically different, some of the percentage differences are highly uncertain. Horizontal lines indicate comparisons using the Mann-Whitney U test: ^****^*P* < 0.0001.

Supplementary Figure 8 Schematics for fluid-coupling calculations. The cross sections of a pair of neighboring stereocilia with radii r_a_ and r_b_ are moving relative to each other with velocity **U** along the line connecting their centers. The gap g(y) between the stereocilia depends on the y-coordinate and has a minimum value g_m_. The distances g_a_ and g_b_ between the y-axis and the stereocilia are used to derive the fluid-coupling force opposing their relative motion.

Supplementary Movie Stereociliary height measurements in representative 3-D reconstructed hair bundles.

Supplementary Movie 1 3D-reconstructed hair bundles from Di4 live imaging – height measurements.

Supplementary Movie 2 3D-reconstructed hair bundles from Phalloidin-Alexa488 mildly-fixed imaging – height measurements.

Supplementary Movie 3 3D-reconstructed hair bundles from FM 4-64FX live imaging – height measurements.

Supplementary Movie 4 3D-reconstructed hair bundles from Di4 live imaging – width measurements.

Supplementary Movie 5 3D-reconstructed hair bundles from FM 4-64FX live imaging – width measurements.

Supplementary Movie 6 3D-reconstructed hair bundles from Phalloidin-Alexa488 mildly-fixed imaging – insertion point separation measurements.
