# Supplementary figures and images for: Dimensions of a Living Cochlear Hair Bundle
Source: Front Cell Dev Biol. 2021 Nov 25;9:742529. doi: 10.3389/fcell.2021.742529 (PMC8657763; doi:10.3389/fcell.2021.742529)

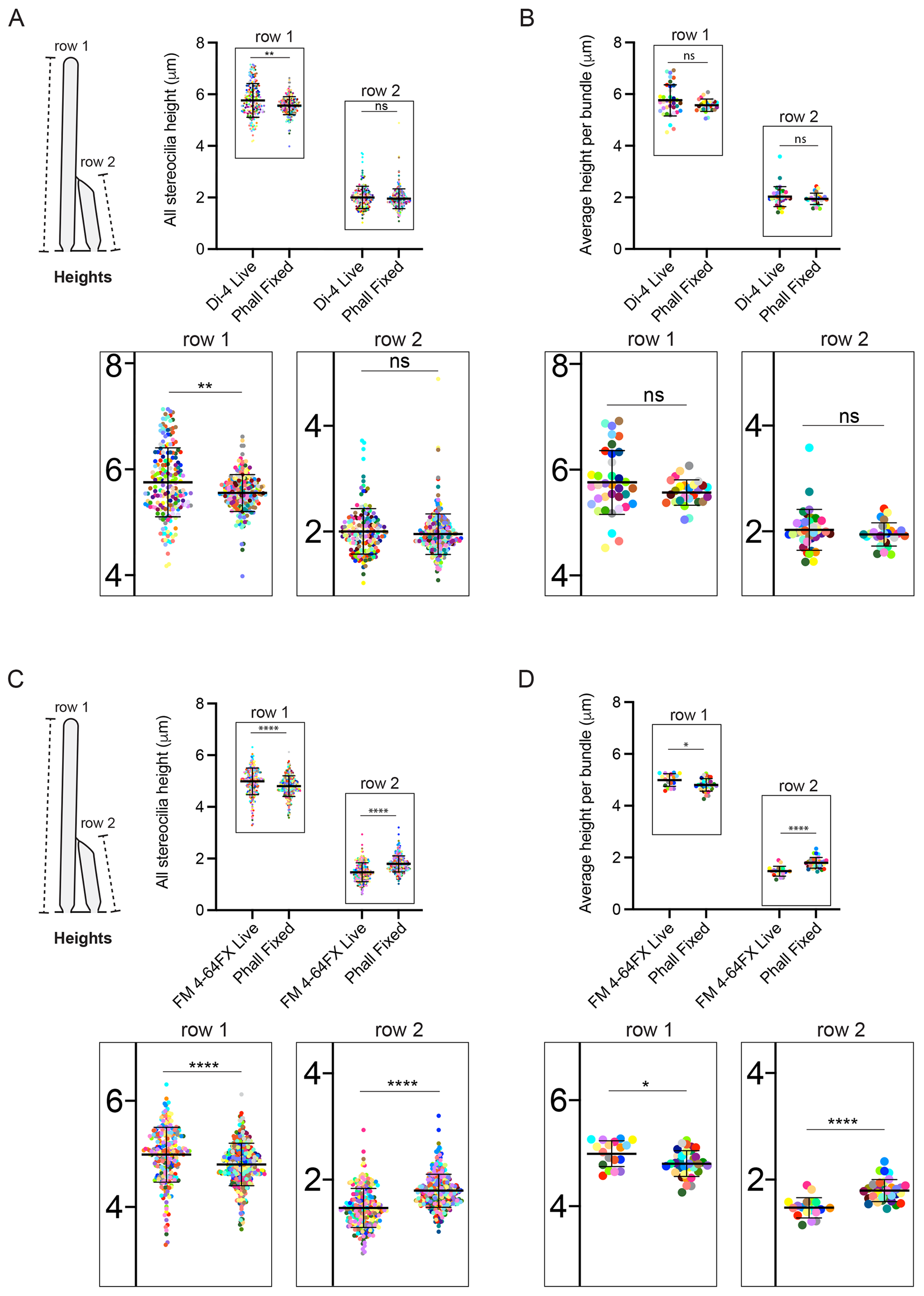

Supplement: Supplementary Figure 1 — Stereociliary heights differ little between live-stained and mildly-fixed conditions. Points of the same color correspond to stereocilia of the same hair bundle, but color schemes differ between conditions and rows. Data is presented at a higher magnification below each plot. (A) Stereociliary heights for all stereocilia are shown from P11 WT IHCs in live Di-4 and mildly-fixed phalloidin conditions. Row 1 Di-4 and phalloidin heights are statistically different, but the percentage difference is small (P = 0.0027, percentage difference = −4 ± 13% relative to Di-4). Row 2 Di-4 and phalloidin heights are not statistically different (P = 0.22). (B) Stereociliary heights averaged per hair bundle are shown from P11 WT IHCs in live Di-4 and mildly-fixed phalloidin conditions. Row 1 Di-4 and phalloidin heights are not statistically different (P = 0.21). Row 2 Di-4 and phalloidin heights are not statistically different (P = 0.32). (C) Stereociliary heights for all stereocilia are shown from P11 WT IHCs in live FM 4-64FX and mildly-fixed phalloidin conditions. Row 1 FM 4-64FX and phalloidin heights are statistically different, but the percentage difference is small (P < 0.0001, percentage difference = −4 ± 13% relative to FM 4-64FX). Row 2 FM 4-64FX and phalloidin heights are statistically different, but the percentage difference is highly uncertain (P < 0.0001, percentage difference = 22 ± 33% relative to FM 4-64FX). (D) Stereocilia heights averaged per hair bundle are shown from P11 WT IHCs in live FM 4-64FX and mildly-fixed phalloidin conditions. Row 1 FM 4-64FX and phalloidin heights are statistically different, but the percentage difference is small (P = 0.017, percentage difference = −4 ± 7% relative to FM 4-64FX). Row 2 FM 4-64FX and phalloidin heights are statistically different, but the percentage difference is highly uncertain (P < 0.0001, percentage difference = 22 ± 19% relative to FM 4-64FX). Horizontal lines indicate comparisons using the Mann-Whitney U test: ns [file Image_1.TIF]

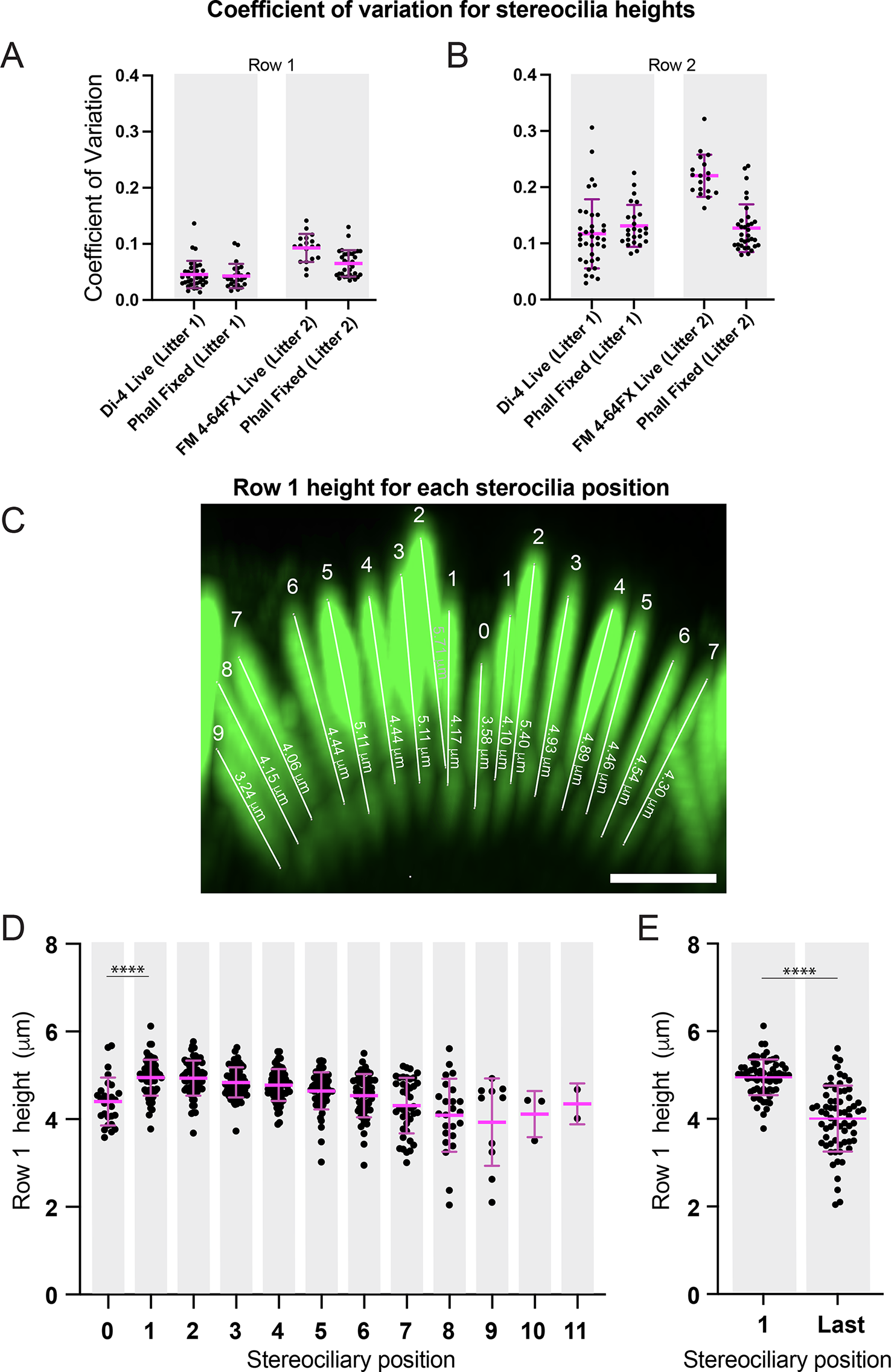

Supplement: Supplementary Figure 2 — Stereociliary height coefficient of variation per hair bundle and stereociliary height relative to row 1 stereociliary position. Coefficients of variation (SD/mean) are shown for row 1 (A) and row 2 (B) stereociliary heights from P11 WT IHCs live-stained with Di-4 or FM 4-64FX or mildly fixed and stained with phalloidin Alexa488 (36 hair bundles, 3 cochleae, 3 animals for Di-4; 25 hair bundles, 3 cochleae, 3 animals for phalloidin; 18 hair bundles, 2 cochleae, 2 animals for FM 4-64FX; 33 hair bundles, 2 cochleae, 2 animals for phalloidin). (C) Row 1 stereocilia are labeled with respect to the column forming the notch, which is at position 0 (Figure 5). An example of a phalloidin-Alexa488 mildly-fixed P11 IHC hair bundle (FM 4-64X) is labeled with the heights of each row 1 stereocilium. Note that the difference in height between the tallest and shortest row 1 stereocilium is more than 2 μm. Scale bars: 2 μm. (D) Row 1 stereociliary heights group by their position within the bundle are shown from phalloidin-Alexa488 mildly-fixed P11 IHCs (33 hair bundles, 2 cochleae, 2 animals) (Litter 2). The phalloidin heights of the position 0 and the position 1 stereocilia are statistically different, but the percentage differences are highly uncertain (stereociliary height: position 0: 4.40 ± 0.54 μm; position 1: 4.95 ± 0.41 μm; percentage difference = −11 ± 14% relative to position 1). (E) The heights of the position 1 and the last stereocilia within a hair bundle wing are compared. The comparison is based on measurements of phalloidin-Alexa488 mildly-fixed P11 IHCs (33 hair bundles, 2 cochleae, 2 animals) (Litter 2). The phalloidin heights of the position 1 and the last position stereocilia are statistically different, but the percentage differences are highly uncertain (stereociliary height: position 1: 4.95 ± 0.41 μm; last stereocilium: 4.01 ± 0.75 μm; percentage difference = −19 ± 17% relative to position 1). Horizontal lines indicate comparisons using the Mann-Whitney U tes [file Image_2.tif]

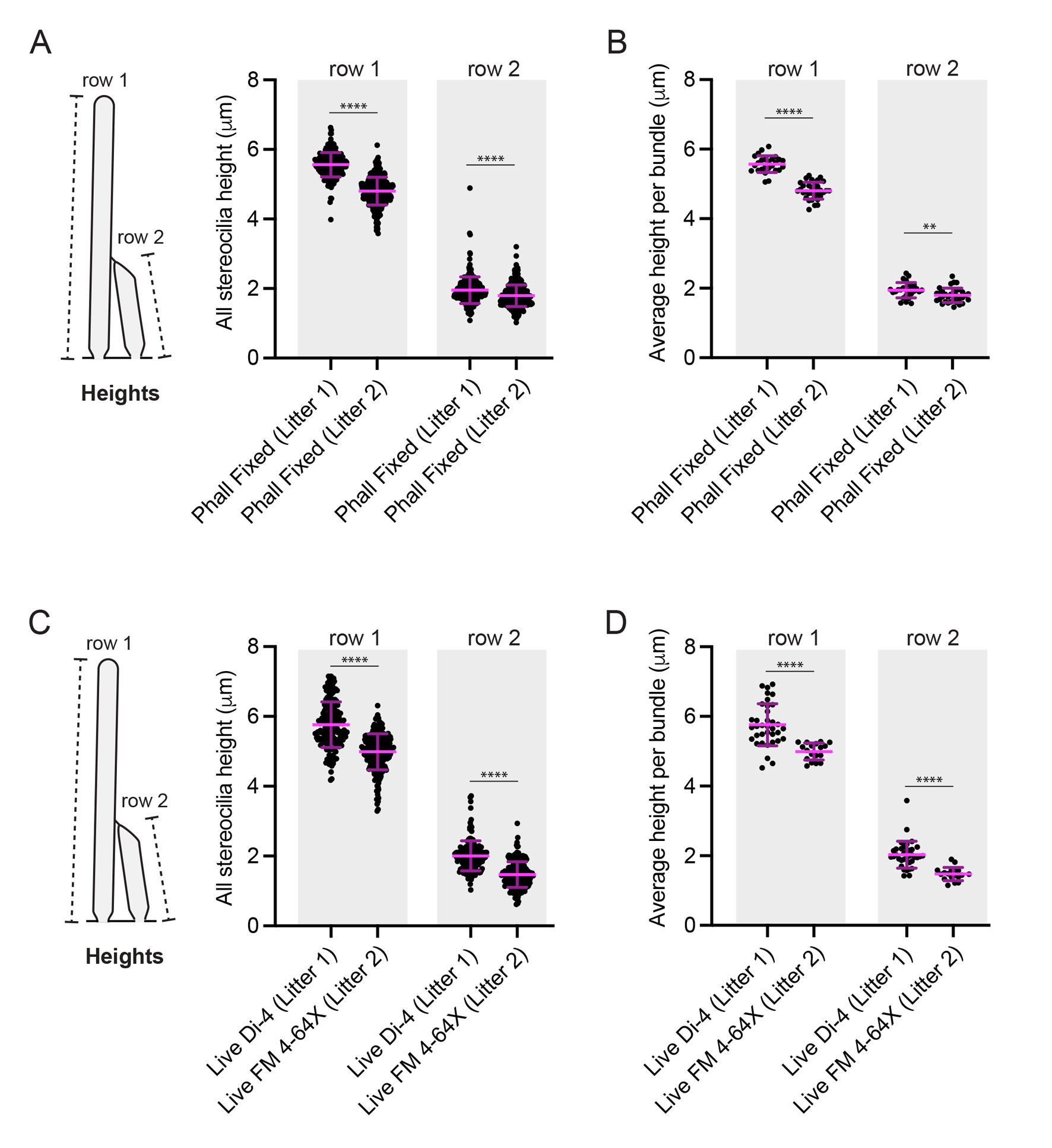

Supplement: Supplementary Figure 3 — Comparisons of stereociliary heights from phalloidin and live-stained conditions. (A) Row 1 but not row 2 stereociliary heights from P11 WT IHCs with phalloidin-labeling in two independent litters are different owing to developmental variability between litters, but the percentage difference is highly uncertain (P < 0.0001 for both row 1 and 2, row 1 percentage difference = −14 ± 10% relative to Litter 1, row 2 percentage difference = −8 ± 25% relative to Litter 1). (B) Average stereociliary height per hair bundle is shown from P11 WT IHCs with phalloidin-labeling in two independent litters. Row 1 and 2 heights are statistically different between the two litters (row 1: P < 0.0001, percentage difference = −14 ± 6% relative to Litter 1; row 2: P = 0.0056, difference = −8 ± 16% relative to Litter 1), but the row 2 percentage difference is too small and uncertain to be biologically important. (C) Stereociliary heights are shown from P11 WT IHCs with live staining in two independent litters (P < 0.0001 both row 1 and 2, row 1 percentage difference = −13 ± 15% relative to Litter 1, row 2 percentage difference = −27 ± 29% relative to Litter 1). Although the heights are statistically different, the percentage differences are highly uncertain. (D) Average stereociliary height per hair bundle is shown from P11 WT IHCs with live staining in two independent litters (P < 0.0001 both row 1 and 2, row 1 percentage difference −13 ± 11% relative to Litter 1, row 2 percentage difference = −27 ± 22% relative to Litter 1). Although the heights are statistically different, the percentage differences are highly uncertain. Magenta lines indicate means ± SDs. Horizontal lines indicate comparisons using the Mann-Whitney U test: ∗∗P < 0.01, ****P < 0.0001. [file Image_3.TIF]

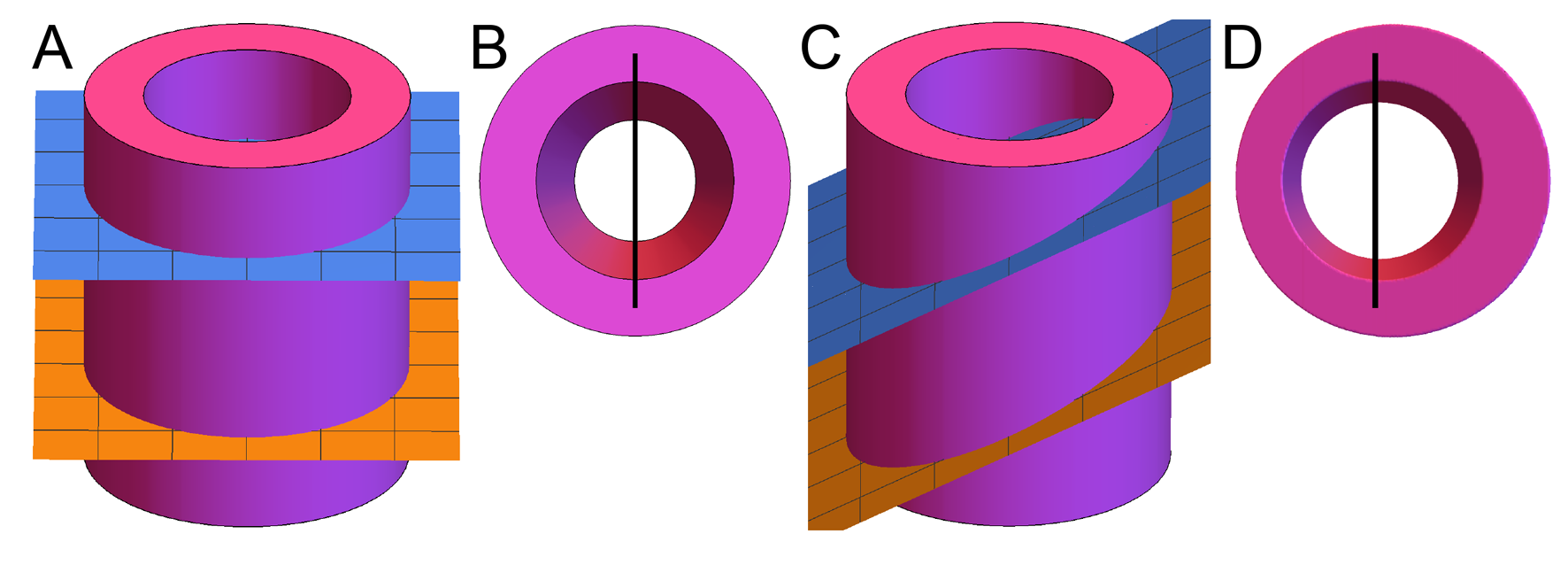

Supplement: Supplementary Figure 4 — The width of a cylindrical stereociliary section must be measured in 3-D. (A) A cylinder’s cross section is shown delimited by two planes perpendicular to a cylinder’s axis. (B) Viewed from the cylinder’s axis, a perpendicular cross section appears circular with a uniform perimeter thickness. The ends of a line (black) used to measure the cylinder’s width are placed on the perimeter’s midsection. The line is chosen to pass though the center of the cylinder’s cavity. (C) A cross section is shown delimited by two planes oblique to the cylinder’s axis. (D) Viewed from the cylinder’s axis, an oblique cross section appears oval with a nonuniform perimeter thickness. The ends of a line (black) used to measure the cylinder’s width are placed on the perimeter’s midsection. The line is chosen to be the shortest line that passes through the center of the cylinder’s cavity. (B,D) Light from planes perpendicular to the cylinder’s axis increase the apparent perimeter thickness in 2-D, which creates error in the determination of the cylinder’s width in 2-D. [file Image_4.TIF]

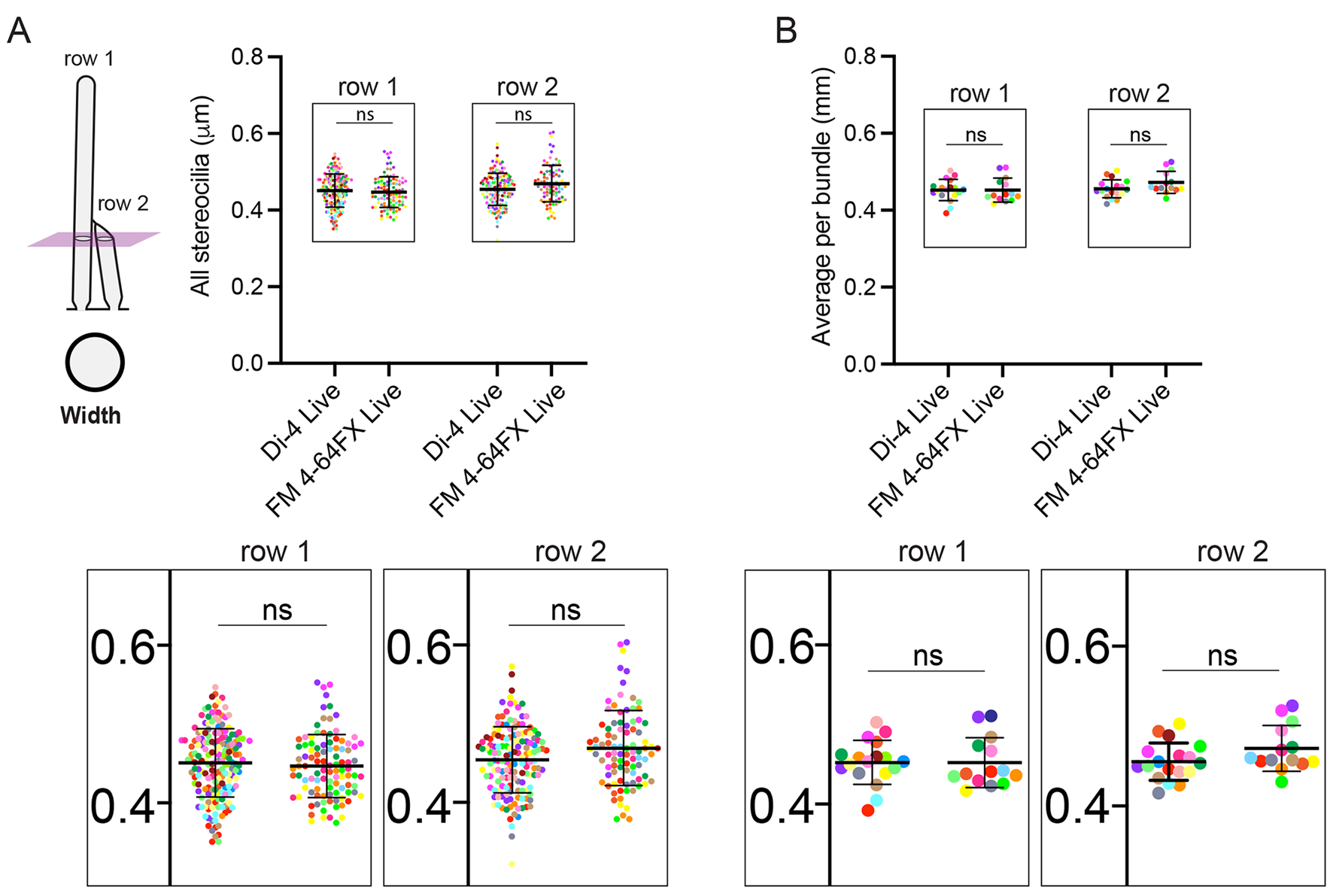

Supplement: Supplementary Figure 5 — Stereociliary widths differ little between Di-4 and FM 4-64FX conditions. Points of the same color correspond to the same hair bundle, but row 1 and 2 color schemes differ between conditions and row. (A) Stereociliary widths are shown from P11 WT IHCs live-stained with Di-4 or FM 4-64FX. Data is presented at a higher magnification below each plot. Row 1 Di-4 and FM 4-64FX widths are not statistically different (P = 0.23). Row 2 Di-4 and FM 4-64FX widths are not statistically different (P = 0.054). (B) Stereociliary widths averaged per hair bundle are shown from P11 WT IHCs live-stained with Di-4 or FM 4-64FX. Row 1 Di-4 and FM 4-64FX widths are not statistically different (P = 0.52). Row 2 Di-4 and FM 4-64FX widths are not statistically different (P = 0.077). Horizontal lines indicate comparisons using the Mann-Whitney U test: ns P > 0.05. [file Image_5.tif]

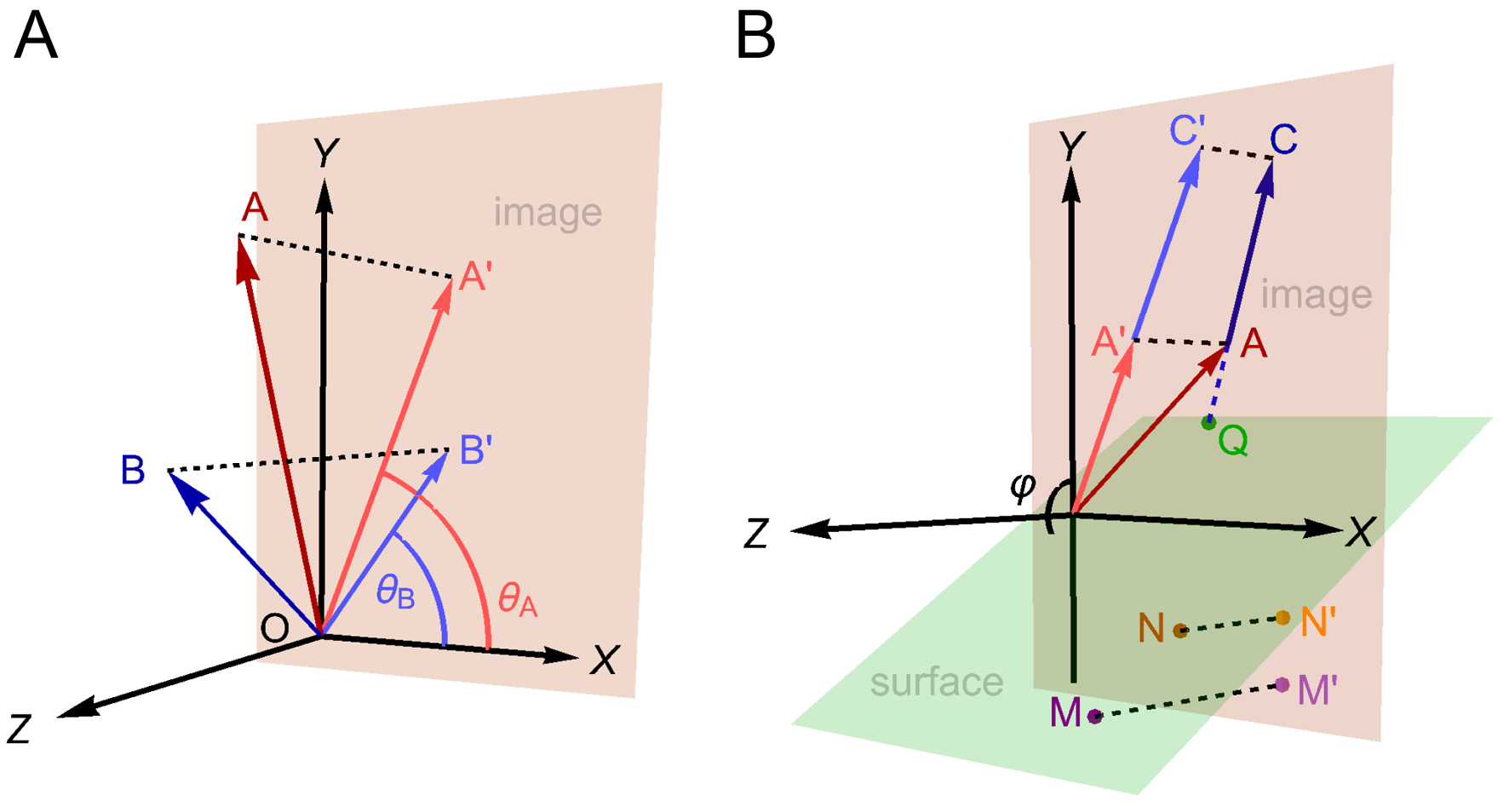

Supplement: Supplementary Figure 6 — Schematics for SEM-height determination from paired images. (A) A stereocilium is represented by the vector (A) in a coordinate frame with an xy-plane coincident with an SEM-image plane. The projection (A)′ onto the image plane is at an angle θA relative to the x-axis. Rotating the sample counterclockwise around the x-axis changes the coordinates such that the stereocilium is now represented by the vector (B). The projection (B)′ onto the image plane is at an angle θB relative to the x-axis. (B) A stereocilium, represented by (A), has a projection (A)′ that obscures the base of a taller stereocilium’s projection onto the image plane. The vector (C)′ represents the visible part of the obscured stereocilium in an SEM image and is the projection of the vector (C) onto the image plane. The apical surface plane of the hair cell is related to the image plane by a counterclockwise rotation though the angle φ . The points N and M on the surface have projections N′ and M′ onto the image plane, such that the line through N′ and M′ is perpendicular to the x-axis. A line through (C) intersects the surface at the point Q . The distance from Q to the point C is the true height of the obscured stereocilium. [file Image_6.TIF]

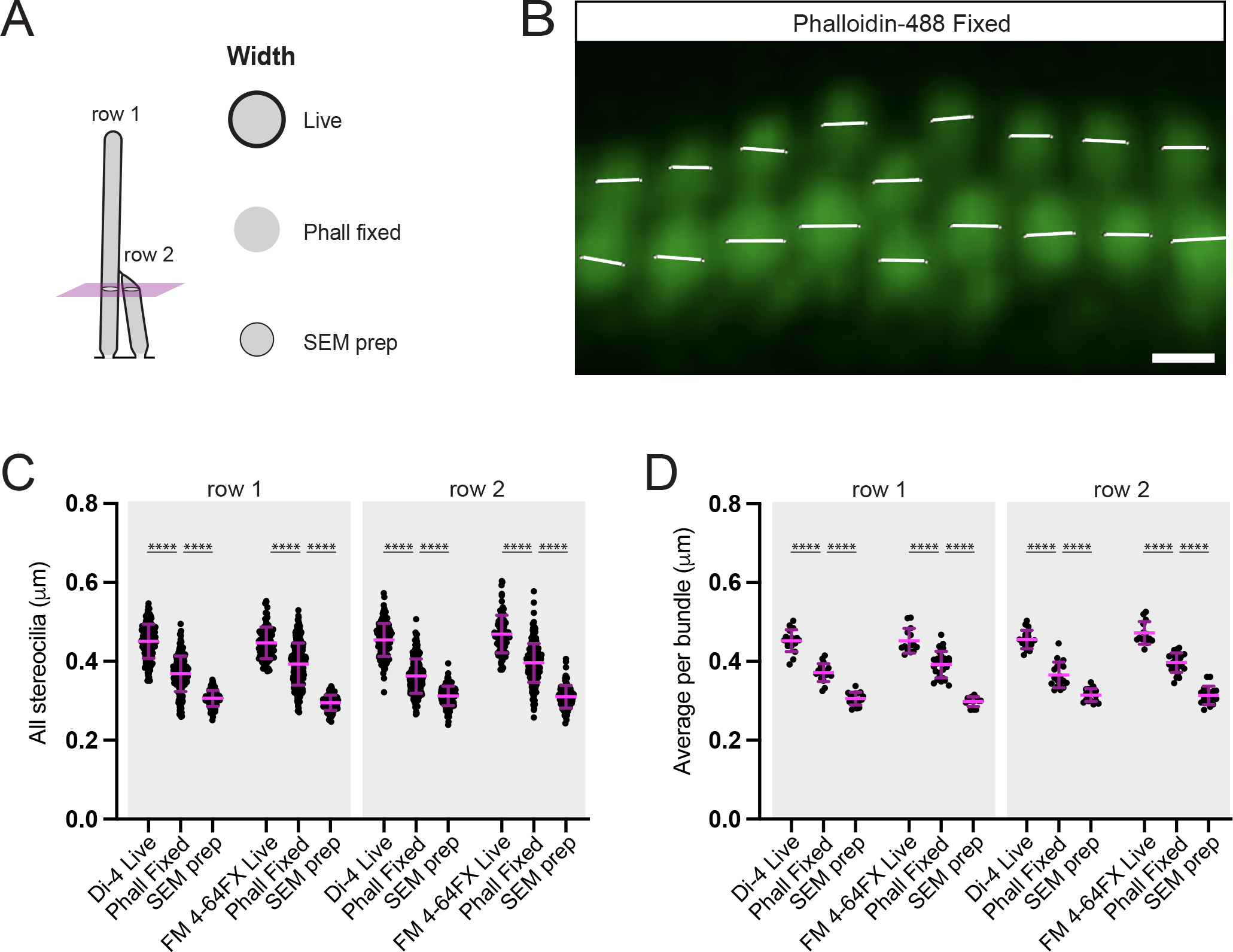

Supplement: Supplementary Figure 7 — Stereociliary width comparison between live, mildly-fixed phalloidin stained, and SEM-prepared samples. (A) A cartoon depicts stereociliary cross sections, upon which width measurements are based, corresponding to different sample-preparation methods. Virtual sections were taken through 3-D reconstructed fluorescently labeled hair bundles below row 2’s stereociliary tips. In Di-4 and FM 4-64FX live conditions, the staining labels the membrane, forming a ring. In phalloidin-488 mildly-fixed samples, the staining labels the actin core ensheathed by the stereociliary membrane, resulting in an filled-circle narrower than the membranous ring. In SEM preparation, the sample shrinks and the membrane is removed by the dehydration procedure. The dehydrated sample is coated with a thin layer of metal for imaging. (B) A representative virtual section trough a phalloidin-488 mildly-fixed hair bundle used for width measurements. Scale bar: 0.5 μm. (C) Stereociliary widths are shown from P11 WT IHCs live-stained with Di-4 (row 1: 172 stereocilia, row 2: 149 stereocilia, 3 cochleae, 3 animals), live-stained with FM 4-64FX (row 1: 101 stereocilia, row 2: 90 stereocilia, 2 cochleae, 2 animals), mildly-fixed with phalloidin (Di-4 litter: row 1: 210 stereocilia, row 2: 189 stereocilia, 3 cochleae, 3 animals) (FM 4-64FX litter: row 1: 318 stereocilia, row 2: 250 stereocilia, 2 cochleae, 2 animals), or prepared for SEM (Di-4 litter: row 1: 119 stereocilia, row 2: 136 stereocilia, 2 cochleae, 2 animals) (FM 4-64FX litter: row 1: 96 stereocilia, row 2: 100 stereocilia, 2 cochleae, 2 animals) (row 1: Di-4 vs. Phall: P < 0.0001, percentage difference = −18 ± 14% relative to Di-4; Di-4-paired Phall vs. SEM: P < 0.0001, percentage difference = −17 ± 14% relative to Phall; FM 4-64FX vs. Phall: P < 0.0001, percentage difference = −12 ± 15% relative to FM; FM-paired-Phall vs. SEM: P < 0.0001, percentage difference = −25 ± 15% relative to Phall; row 2: Di-4 vs. Phall: P < 0.0001, percentage diffe [file Image_7.tif]

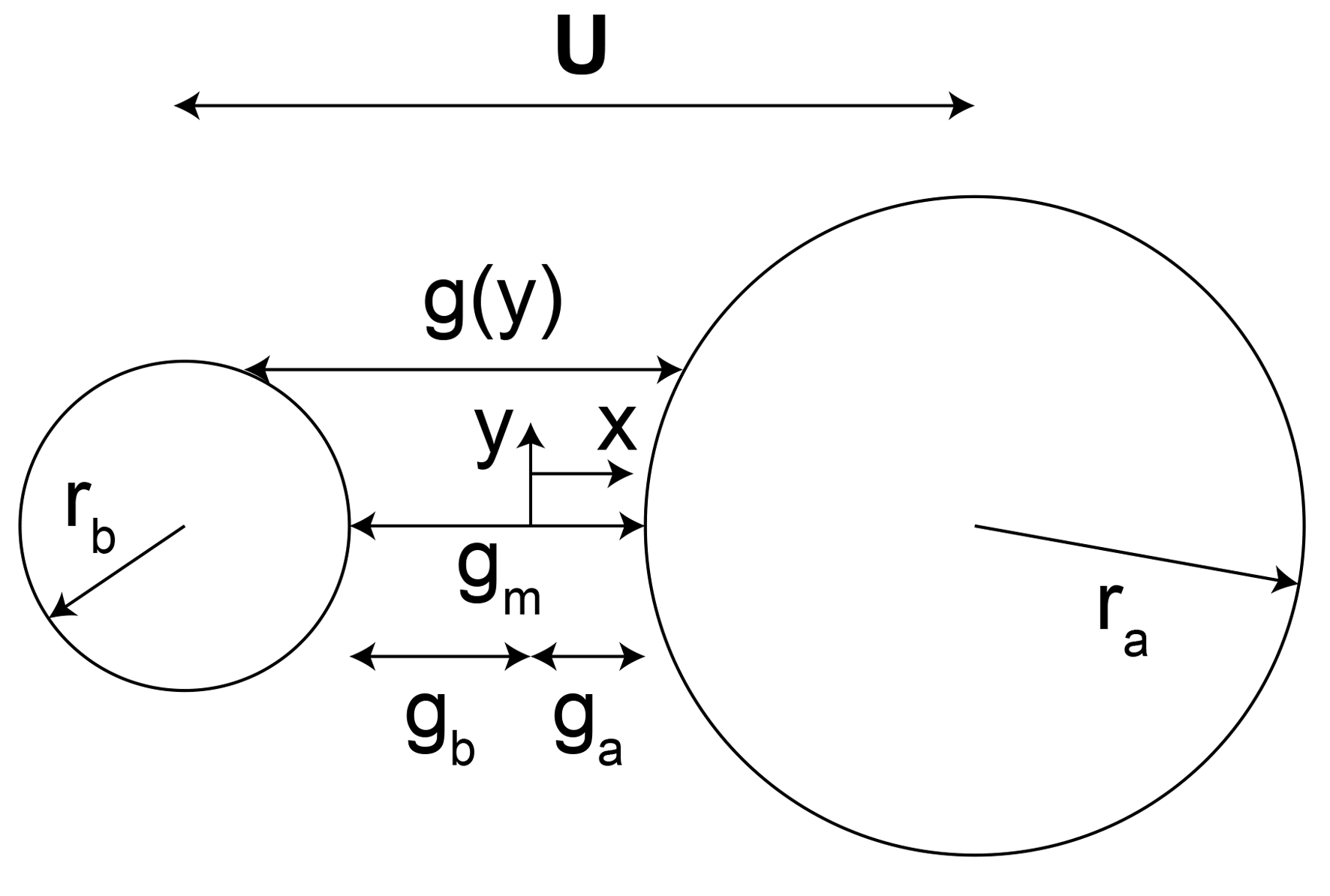

Supplement: Supplementary Figure 8 — Schematics for fluid-coupling calculations. The cross sections of a pair of neighboring stereocilia with radii ra and rb are moving relative to each other with velocity U along the line connecting their centers. The gap g(y) between the stereocilia depends on the y-coordinate and has a minimum value gm. The distances ga and gb between the y-axis and the stereocilia are used to derive the fluid-coupling force opposing their relative motion. [file Image_8.TIF]
